# A natural allele of the transcription factor gene *TaMYB-D7b* is a genetic signature for phosphorus deficiency in wheat

**DOI:** 10.1093/plphys/kiaf224

**Published:** 2025-07-16

**Authors:** Daijing Zhang, Min Fan, Tian Li, Yahya Rauf, Yongjie Liu, Xinkai Zhu, Haiyan Jia, Wenxuan Zhai, Juan C Luzuriaga, Brett F Carver, Liuling Yan

**Affiliations:** Department of Plant and Soil Sciences, Oklahoma State University, Stillwater, OK 74078, USA; College of Life Sciences, Henan Normal University, Xinxiang, Henan 453007, China; Department of Plant and Soil Sciences, Oklahoma State University, Stillwater, OK 74078, USA; Department of Plant and Soil Sciences, Oklahoma State University, Stillwater, OK 74078, USA; State Key Laboratory of Crop Gene Resources and Breeding, Institute of Crop Sciences, Chinese Academy of Agricultural Sciences, Beijing 100081, China; Department of Plant and Soil Sciences, Oklahoma State University, Stillwater, OK 74078, USA; Amarillo Research and Extension Center, Texas A&M AgriLife Research, Amarillo, TX 79106, USA; Department of Plant and Soil Sciences, Oklahoma State University, Stillwater, OK 74078, USA; Department of Plant and Soil Sciences, Oklahoma State University, Stillwater, OK 74078, USA; Agricultural College, Yangzhou University, Yangzhou, Jiangsu 225009, China; Department of Plant and Soil Sciences, Oklahoma State University, Stillwater, OK 74078, USA; The Applied Plant Genomics Laboratory, National Key Laboratory of Crop Genetics and Germplasm Enhancement, Nanjing Agricultural University, Nanjing, Jiangsu 210095, China; Department of Plant and Soil Sciences, Oklahoma State University, Stillwater, OK 74078, USA; Department of Plant and Soil Sciences, Oklahoma State University, Stillwater, OK 74078, USA; Department of Plant and Soil Sciences, Oklahoma State University, Stillwater, OK 74078, USA; Department of Plant and Soil Sciences, Oklahoma State University, Stillwater, OK 74078, USA

## Abstract

Phosphorus (P) and nitrogen (N) deficiencies are major yield-limiting factors for wheat (*Triticum aestivum*) production worldwide, particularly in the acidic soils of the southern Great Plains of North America. In this study, we report that the transcription factor gene *TaMYB-D7* is responsible for a major quantitative trait locus controlling purple leaf color. The wheat cultivar “2174” showed purple coleoptiles regardless of P status, whereas “Jagger” did not, even under P limitation. The 2 cultivars differed by 1 amino acid in *Ta*MYB-D7: Gly-50 in *Ta*MYB-D7b (encoded by the 2174 allele) and Ser-50 in *Ta*MYB-D7a (encoded by the Jagger allele). We used genome editing to inactivate all 3 *TaMYB7* homoeologs in cv. 2174. The resulting edited wheat plants did not accumulate purple pigments throughout their life cycle, validating the functions of *TaMYB-7Db* associated with the purple phenotype. In the *TaMYB7*-edited plants, *chalcone synthase 2-like* (*TaCHSL2*), which may be involved in anthocyanidin biosynthesis and metabolism, was dramatically downregulated, suggesting that *Ta*MYB7 induces its transcription. We also discovered that the expression of *TaMYB7* and *TaCHSL2* was upregulated by P but downregulated by N. Lastly, we developed a Kompetitive Allele Specific PCR (KASP) marker to facilitate the genotyping of *TaMYB-D7b*, which can be used for marker-assisted breeding. Our results provide insight into nutrient use efficiency in wheat.

## Teaser

A naturally occurring gene variant changes leaf pigmentation in response to phosphorus availability and offers a visible marker for nutrient status in common wheat.

## Introduction

Nitrogen (N) and phosphorus (P) are 2 of the essential macronutrients needed to sustain plant growth and development and enable plants to complete their life cycle (Santi et al. 2013; Nadeem et al. 2022). P is a major limiting factor in crop production worldwide because approximately 50% of agricultural soils are acidic (Kochian et al. 2004; Yan et al. 2006; Raman and Gustafson 2014). P deficiency is particularly severe in the southern Great Plains of North America, where acidic soils with pH <5.0 are predominant (Carver et al. 1988; Liu et al. 2015). In acidic soils, inorganic P is bound to aluminum and iron oxides, which are fixed into organic forms, resulting in low P bioavailability for crops (Carver et al. 1993; Raghothama 1999; Yan et al. 2006; Nadeem et al. 2022). N and P fertilizers have become one of the most critical additions yearly to support crop production. However, the high P-fixing capacity of soils results in low uptake and utilization by crop plants, with usage rates <20% for fertilized P and only 30% to 35% for added N fertilizers each year, while the remainder contributes to nutrient pollution of the environment (McLaughlin et al. 1988; Raun and Johnson 1999; Gaju et al. 2011; McBeath et al. 2012; Zhu et al. 2013; Gong et al. 2016).

The most striking symptom of P deficiency is the accumulation of a purple pigment in leaves and other symptoms such as dark green leaves, slower growth, and later maturity (Batten 1992). In several crop species, purple pigmentation is consistently observed under diverse environmental conditions at certain growth phases. These phenotypes are easily scored with the naked eye and are used as a classical phenotypic marker. However, the purple color can also be caused by a lack of N supply in soils or by other factors, such as aluminum toxicity and cold weather, making it difficult to distinguish between its possible causes (Zhu et al. 2013).

Purple pigmentation in plants usually reflects the accumulation of anthocyanins, pigments from the flavonoid family that can give red, blue, and purple colors to vegetative and nonvegetative plant tissues (Tanaka et al. 2008; Alappat and Alappat 2020). Anthocyanins are involved in multiple plant functions, such as attracting pollinators, low-temperature resilience, and protection against ultraviolet light and pathogen attacks (Field et al. 2001; Schaefer et al. 2004; Lotkowska et al. 2015; Liu et al. 2018a, Liu et al. 2018b). Anthocyanins have been intensively studied due to their antioxidant properties and protective benefits for human health, including against cancer, inflammation, and heart disease (He et al. 2011; Khoo et al. 2017; Alappat and Alappat 2020). The common flavonoid biosynthetic pathway involves anthocyanin and proanthocyanidin biosynthesis, which relies on 5 biosynthetic enzymes (Winkel-Shirley 2001). The genes encoding these enzymes are classified as early flavonoid biosynthetic genes, such as *Chalcone synthase* (*CHS*), and late flavonoid biosynthetic genes, such as *Chalcone flavanone* (*CHI*) (Li 2014). However, it has not been possible to determine whether the purple color in a given plant is caused by a P deficiency or genetic factors affecting purple pigmentation.

Wheat (*Triticum aestivum* L. 2*n* = 6*x* = 42; AABBDD genome) is sensitive to macronutrient deficiencies, primarily of N and P (Manske et al. 2001). An imbalance in the ratios of these 2 macronutrients in a given genotype may result in a characteristic accumulation of purple pigments in different wheat tissues at various developmental stages, making it challenging to identify which nutrient is lacking and responsible for the change in color (Zhu et al. 2013). Wheat has numerous genes controlling anthocyanin accumulation. The genes underlying pigmentation for anthers (*Pan*), culms (*Pc*), glumes (*Pg*), leaf blades (*Plb*), leaf sheaths (*Pls*), pericarp (*Pp*), grains (*R*), auricles (*Ra*), and coleoptiles (*Rc*) are reported in the gene catalog for wheat. The pigmentation genes *Rc, Pc, Pan, Plb, and Pls* are closely linked, residing on the short arms of homoeologous group 7 chromosomes (Jha 1964; Michael and Richard 1971; Sutka 1977; Khlestkina et al. 2002, 2010). Among them, only the *R* gene has been cloned, and it was shown to encode a Myeloblastosis (MYB) transcription factor that regulates proanthocyanidin biosynthesis (Himi and Taketa 2015). The purple color of the coleoptile in wheat is genetically determined by 2 dominant genes, *Rc3* and *Rc4*, located on chromosomes 7D and 6B, respectively (Sutka 1977). In addition, the *Rc-A1b* (red coleoptile) gene located on chromosome 7A is associated with the repression of *F3h-1* encoding flavanone 3-hydroxylase expression and anthocyanin pigmentation in the coleoptiles of Russian bread wheat (Khlestkina et al. 2010).

In this study, we cloned a gene from wheat responsible for purple pigment accumulation in the cultivar “2114”. A single-nucleotide polymorphism (SNP) replacing Ser-50 with a Gly residue in the MYB transcription factor *Ta*MYB-D7 explained the phenotypic variation between 2 wheat cultivars differing in the pigmentation of their leaves. Notably, the *TaMYB-D7b* allele associated with the purple color can be used as a genetic signature for P in wheat and minimize other possible environmental factors resulting in purple pigment accumulation.

## Results

### Regulation of purple color by P and N nutrition in 2 winter wheat cultivars

**Figure 1.**
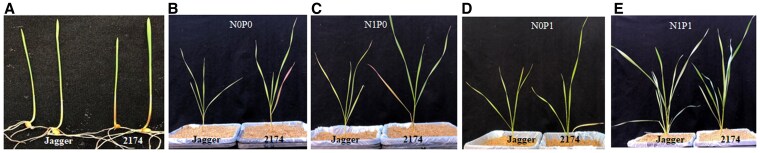
Phenotypes at the seedling stage. **A)** Representative photographs of coleoptiles from Jagger and 2174 seedlings. Seeds from the 2 parental lines were germinated on paper towels soaked with water without any nutrients. **B** to **E)** Representative photographs of Jagger and 2174 plants tested in sandy soil without N or P **B)** with N but without P **C)** without N but with P **D)** and with N and P **E)**.

In a previous study, we found that 2 winter wheat cultivars, cv. Jagger and cv. 2174, had different responses in coleoptile and leaf colors to N and P deficiency (Zhu et al. 2013). In this study, we tested the 2 cultivars on water-soaked paper towels in Petri plates or sandy soil with precisely defined fertilizer treatment. When germinated on plates without added nutrients, the base of cv. 2174 coleoptiles turned light purple, whereas Jagger seedlings had colorless coleoptiles (Fig. 1A). This result indicates that the purple color is controlled by gene(s) in cv. 2174 or that the absence of purple is influenced by gene(s) in Jagger. In plants grown in sandy soil without any N or P for 4 wk, the entire blade of the 1st leaf from cv. 2174 plants turned purple, contrasting with the yellow color seen for Jagger plants (Fig. 1B). We observed the same purple color on the 1st leaf of cv. 2174 plants grown in the absence of P (Fig. 1C) but not in the absence of N (Fig. 1D). When provided with sufficient N and P nutrients, the 1st leaf of cv. 2174 plants did not accumulate purple pigments (Fig. 1E). Under the same conditions, Jagger plants never showed any purple leaf color (Fig. 1, D and E). We conclude that the purple coleoptile color in cv. 2174 is genetically controlled but can be eliminated by supplying adequate P. Notably, Jagger does not have an active allele of the gene responsible for producing the purple color in seedlings.

**Figure 2.**
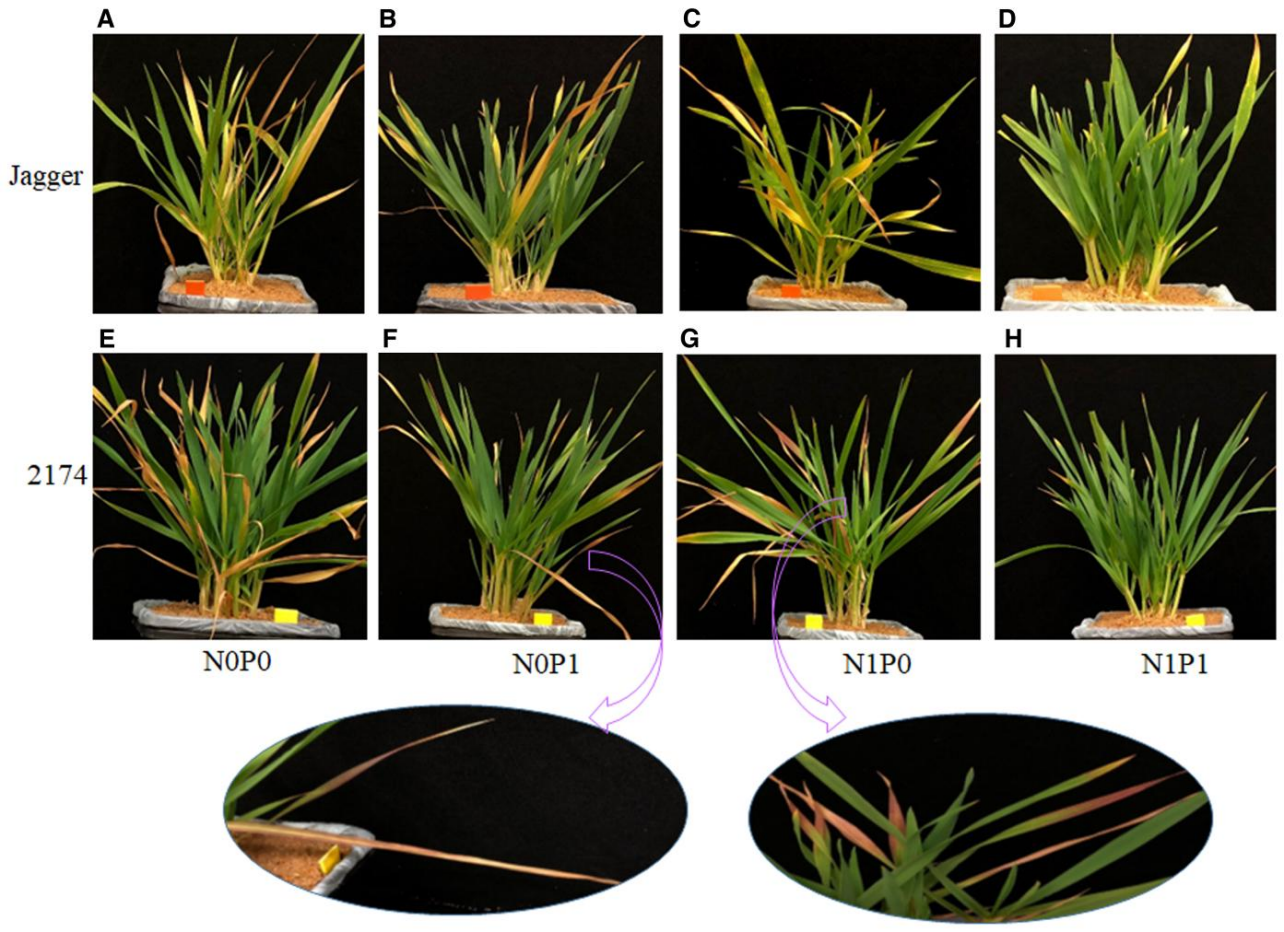
Phenotypes of Jagger and 2174 plants grown in sandy soil. Jagger **A** to **D)** and 2174 **E** to **H)** plants were grown in sandy soil for 14 wk, after which nutrient supply was withheld. The photographs were taken 1 wk later. **A** and **E)** plants lacking N (0) and P (0). **B** and **F)** plants lacking N (0) but with full P nutrition (1). **C** and **G)** plants lacking P (0) but with full N nutrition (1). **D** and **H)** plants with full N (1) and P nutrition (1). All images were taken at the same scale. Arrows indicate the purple color developing on 2174 leaves under N deficiency **F)** or P deficiency **G)**. The oval images shown at the bottom of the figure have been featured from the same images as shown in **F)** and **G)**.

When grown in sandy soil with adequate N and P for 14 wk, neither cv. 2174 nor cv. Jagger plants had young or old leaves of purple color. We determined that the accumulation of purple pigments is conditional and depends on low P supply, as we could induce the purple phenotype in healthy plants by withholding P for 1 wk. Indeed, starting with healthy plants, we withheld N and P (Fig. 2A), N only (Fig. 2B) or P only (Fig. 2C) for 1 wk, at which point some of the old leaves of Jagger turned yellow, burned, and then withered, while Jagger plants remaining on full N and P supply stayed green (Fig. 2D). However, cv. 2174 leaves looked burned and withered when both N and P were withheld (Fig. 2E), turned slightly purple from the tips of young leaves when no N was supplied (Fig. 2F), and had a strong purple color in the young leaves when no P was supplied (Fig. 2G), in contrast to the healthy green seen with cv. 2174 plants on full N and P supply (Fig. 2H). These results indicate that cv. 2174 is highly sensitive to N and P deficiency, which results in the production of purple pigments.

### Identification of a quantitative trait loci associated with purple leaves in wheat

**Figure 3.**
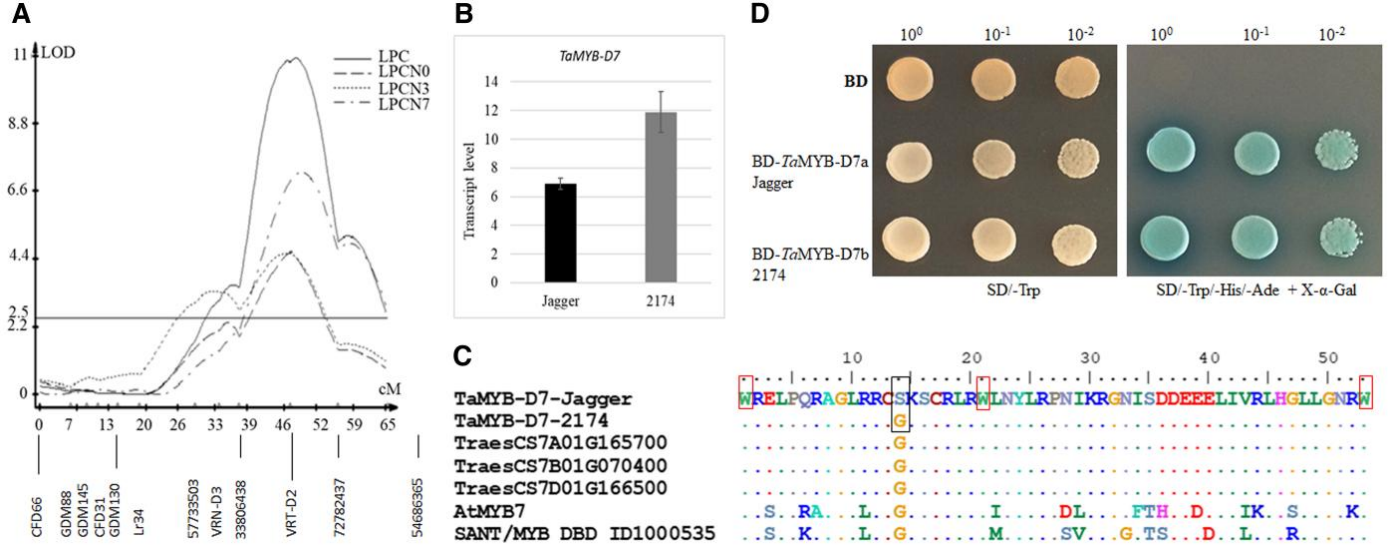
Mapping of *QPc.osu-7D* and characterization of *Ta*MYB-D7 alleles from 2174 and Jagger. **A)** QTL mapping of *QPc.osu-7D*. The RIL population was tested on commercial soil but not supplied with any N or P fertilizer. The first population was phenotyped twice, first on January 30 (LPCN0) and again on April 25, 2008 (LPCN3). The same set of RILs was tested in yellow soil lacking N. The second population was also phenotyped twice, first on December 25, 2008 (LPCN) and again on January 27 2009 (LPCN7). The phenotypic data were integrated with genotypes for QTL mapping. The Kosambi mapping function was used to estimate the map distance using MapMaker 3.0. The Interval Mapping function was used to locate QTLs with WinQTL Cart 2.5. Molecular markers around the *QPc.osu-7D* locus were mapped in a genetic distance (cM). **B)** Relative transcript levels of *Ta*MYB-D7a from Jagger and *Ta*MYB-D7b in 2174 as determined by qPCR, using *Actin* as a reference transcript. The gene transcript levels were calculated using the 2^−ΔΔCT^ method, where CT is the threshold cycle. Values are means ± standard error (*n* = 9). **C)** Multiple sequence alignment of the conserved 53 amino acids in *Ta*MYB-D7, its homoeologs from hexaploid wheat, and homolog from Arabidopsis. Three tryptophan (Trp, W) residues in the HTH sequence are highlighted with red squares. The Ser-50-Gly substitution between *Ta*MYB-B7a and *Ta*MYB-D7b is highlighted with a black square. **D)**  *Ta*MYB-D7 auto-activates transcription in a yeast assay. The coding sequence of *Ta*MYB-D7a from Jagger and that of *Ta*MYB-D7b from 2174 were cloned in-frame with a yeast vector containing the sequence of the yeast GAL4 DNA-BD. Transformed cells were grown on a synthetic defined (SD) medium lacking Trp (SD/–Trp) and on an SD medium lacking Trp, His, and Ade (SD/−Trp/−His/−Ade). Three 10-fold dilutions from each yeast colony were spotted on the same plates.

To determine whether the same genes control the accumulation of purple pigments in coleoptiles and leaves and whether they derive from one or both cv. Jagger and cv. 2174, we tested a recombinant inbred line (RIL) population from a cross between the 2 cultivars in the commercial soil without added N or P. The 144 RILs were genotyped using SSR markers (Chen et al. 2010) and SNP markers (Li et al. 2013). We integrated the phenotype collected for leaf color with the SNP data to map quantitative trait loci (QTLs). We detected 1 major QTL on chromosome 7D (*QPc.osu-7D*) that is associated with the purple color of leaves (Fig. 3A). The logarithm of the odds (LOD) score of *QPc.osu-7D* was 4.6 to 10.9, accounting for 20.2% to 44.6% of the total phenotypic variation in the same set of populations. We assembled 21 SNP markers into a group that included 10 linked simple sequence repeat (SSR) markers and 3 PCR markers for the functional genes on chromosome 7D, including *Leaf rust resistance gene 34* (*Lr34-D1*) (Cao et al. 2010), *Vernalization gene 3* (*VRN-D3*) (Chen et al. 2010), and *Vegetative to reproductive transition gene 2* (*TaVRT-D2*) (Xie et al. 2021). These results indicate the presence of a major gene associated with purple leaves on the short arm of chromosome 7D.

### Allelic variation in TraesCS7D02G166500

The *QPc.osu-7D* covered an interval of about 20 cM on chromosome 7D (Fig. 3A). Candidate genes for *QPc.osu-7D* were found to be located in the genomic region between 2 markers, SNP 33806438 and *VRT-D2*, based on the crossovers in 4 RILs (13, 14, 37, and 66) in the mapping population. There are 292 genes from TraesCS7D02G151700 to TraesCS7D02G176700 annotated with high confidence in the candidate gene region. Since the targeted region exhibits low chromosomal recombination (Cao et al. 2010) and genome complexity due to large chromosomal duplications (Fang et al. 2020), fine mapping was not feasible. Given these constraints, we prioritized candidate genes based on their known functional relevance to pigment accumulation. Among the 292 genes within the targeted QTL region, TraesCS7D02G166500, annotated as encoding a MYB domain transcription factor in the Chinese Spring (CS) reference genome (IWGSC 2018), was identified as a good candidate for *QPc.osu-7D*. Our selection was guided by the established role of MYB transcription factors in flavonoid synthesis and pigment accumulation. While other genes within the QTL region may also contribute to flavonoid biosynthesis through MBW complexes (MYB-bHLH-WD40) or alternative regulatory pathways in plant species (Xu et al. 2021), we focused on TraesCS7D02G166500 for further investigation. The pigmentation genes *Rc, Pc, Pan, Plb*, and *Pls* are closely linked, and most of them reside on the short arms of homoeologous group 7 chromosomes (Jha 1964; Michael and Richard 1971; Sutka 1977). The 3 genes on each homoeologous group 7 chromosome have been designated as 7A (*Rc-A1*), *7B* (*Rc-B1*), and *7D* (*Rc-D1*) (Khlestkina et al. 2002), *TaC1-A1*, *TaC1-B1*, and *TaC1-D1* (Himi and Taketa 2015), and *TaMYB-A1, TaMYB-B1, and TaMYB-D1* (Cao et al. 2019). Although these previous studies reported that these genes are associated with purple coleoptiles, whether they show allelic variation among cultivars has not been investigated.

TraesCS7D02G166500 protein shows the highest percentage identity with the transcriptional repressor MYB7 (61% similarity) in Arabidopsis (*Arabidopsis thaliana*) (GenBank accession number OAP08362). We, therefore, renamed TraesCS7D02G166500 as *TaMYB-D7*. We looked for polymorphisms in *TaMYB-D7* between Jagger and 2174 by isolating the complete *TaMYB-D7* gene, sequencing the 2 alleles, and comparing the resulting sequences to that from CS. *TaMYB-D7* in CS is 2,865 bp in length from the ATG to the stop codon, with 3 exons and 2 introns (Supplementary Fig. S1). We determined that *TaMYB-D7* from Jagger and 2174 has 2 exons and 1 intron of only 880 bp (Supplementary Fig. S1). To be consistent with the names of alleles in other genes in Jagger and 2174 from previous studies (Li et al. 2013), we designated the Jagger allele as *TaMYB-D7a* and the 2174 allele as *TaMYB-D7b*. We used *TaMYB-D7c* for the CS allele.

*TaMYB-D7a* and *TaMYB-D7b* differed in leaf transcript levels, with *TaMYB-D7* being expressed about twice as strongly in 2174 as in Jagger (Fig. 3B). However, this relatively modest difference in expression levels was difficult to reconcile with the binary response of the 2 cultivars, suggesting that the encoded proteins might differ in their activity and cause the difference in phenotype. Based on the above sequencing, we identified a single SNP in exon 1 between the *TaMYB-D7a* and *TaMYB-D7b* alleles, changing Gly-50 in *Ta*MYB-D7b to Ser in *Ta*MYB-D7a (Fig. 3C). MYB proteins share a conserved DNA-binding domain (BD) with a helix-turn-helix (HTH) structure (Ogata et al. 1992). In plant MYB proteins, this domain generally comprises 2 or 3 imperfect repeats, called the R2R3-type domain (Stracke et al. 2001), with each repeat taking on the HTH structure and comprising about 53 amino acids. These 3 typical HTH structures are interspersed with tryptophan (Trp, W) residues, forming a tryptophan cluster. *Ta*MYB-D7 is an R3-type MYB with 3 W residues located at positions 36, 57, and 89 in *Ta*MYB-D7 (Fig. 3C, Supplementary Fig. S2). The Ser-50-Gly polymorphism occurred in the helix (Fig. 3C) but did not affect the auto-activation potentials of the 2 proteins when tested in a yeast transactivation assay (Fig. 3D). Therefore, *Ta*MYB-D7a and *Ta*MYB-D7b do not differ in their transcriptional activation potential. We speculate that these 2 proteins may vary in their DNA-binding specificity and/or interactions with other proteins.

### *TaCHSL2* genes were regulated by *Ta*MYB-D7

**Figure 4.**
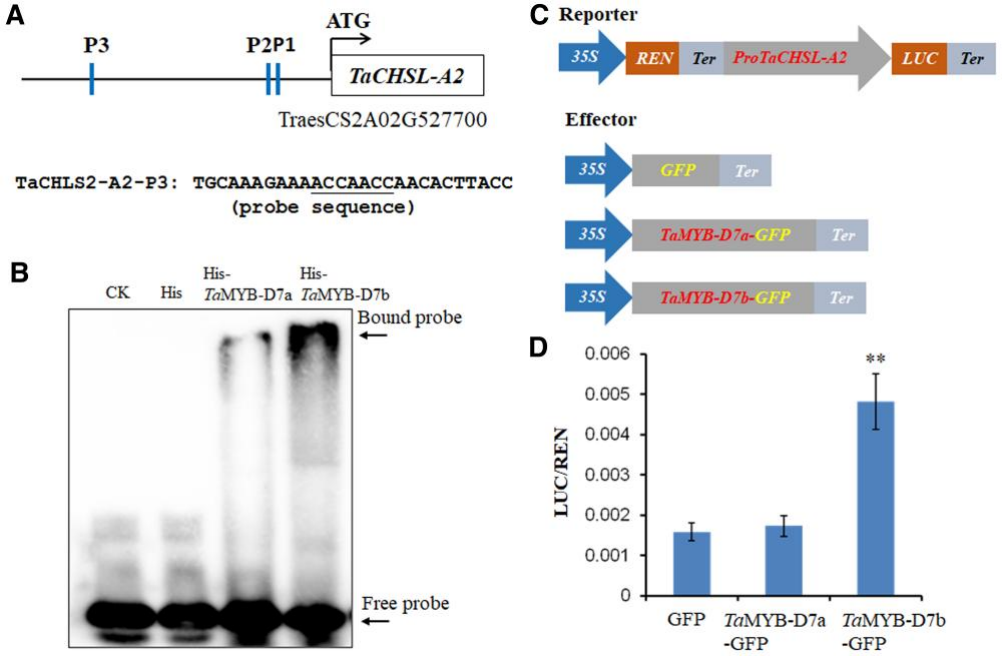
Protein expression and EMSA assay. **A)** DNA probe for EMSA. The underlined sequence is the DNA-binding site in the promoter of *Ta*CHSL-A2. **B)** Interactions of *Ta*MYB-D7 with the DNA probe in EMSA assay in vitro. *Ta*MYB-D7b (from 2174) exhibited higher binding affinity compared with *Ta*MYB-D7a (from Jagger). His tag alone was used as a negative control in the assay. **C)** Schematic diagram of the reporters and effectors used in the transient assays. Firefly luciferase (LUC); Renilla luciferase (REN). **D)** Transient expression assay in *N. benthamiana* leaves showing that *Ta*MYB-D7b (from 2174) activated the transcription of *Ta*CHSL-A2, while the empty control and *Ta*MYB-D7a (from Jagger) showed inactivation. Relative LUC activity (LUC/REN) was measured at 72 h after infiltration. Values are presented as mean ± SD of 3 independent replicates. ** indicates significant level in *t*-test (*P* < 0.01).

The *TaCHSL2* genes are represented by 3 homoeologs on chromosome 5 (TraesCS2A02G527700, TraesCS2B02G558400, and TraesCS2D02G530600) and encode CHS 2-like proteins. The predicted proteins show a high degree of identity to CHS involved in the biosynthesis of flavonoids and floral pigments (Hahlbrock and Scheel 1989; Dao et al. 2011). We detected 5 potential DNA-binding motifs (5′-ACCTAC-3′, 5′-GGATGGT-3′, 5′-GGTAGGT-3′, 5′-CCTACC-3′, and 5′-ACCTAAC-3′) in the promoter regions within ∼400 bp upstream of the ATG start codon in each of the 3 *TaCHSL2* homoeologs (Supplementary Fig. S3). These potential DNA-binding motifs are similar to CC(A/T)ACC, which is recognized by maize (*Zea mays*) MYB-like protein (Grotewold et al. 1994), and to several DNA motifs recognized by Arabidopsis MYB proteins, such as 5′-ACCTACCA-3′ by MYB6 and MYB7, 5′-ACCTACC-3′ by MYB63 and MYB85, and 5′-ACCAACC-3′ by MYB78 (Wang et al. 2020). We identified multiple *Ta*MYB-D7 binding sites in the promoter region of *TaCHSL-A2* (Supplementary Fig. S3). We selected a 27-bp fragment containing ACCAACC in the *Ta*CHSL-A2 promoter that is predicted to be bound by *Ta*MYB-D7 (Fig. 4A). We expressed and purified the *Ta*MYB-D7 proteins in *E. coli*. We conducted an electrophoretic mobility shift assay (EMSA) to assess whether *Ta*MYB-D7 directly binds to the DNA element. The EMSA results showed that *Ta*MYB-D7b (from 2174) had a much stronger binding affinity with the DNA element in the *Ta*CHSL-A2 promoter compared with *Ta*MYB-D7a (from Jagger) (Fig. 4B), indicating that a single amino acid substitution between *Ta*MYB-D7b and *Ta*MBY-D7a proteins plays a crucial role in determining the binding activity of *Ta*MYB-D7 proteins in wheat.

We also conducted transient expression assays to investigate that *Ta*MYB-D7a and *Ta*MYB-D7b have different activities to activate the transcriptions of the downstream gene *TaCHSL-A2*. We constructed a *Ta*MYB-D7 effector driven by the 35S promoter, along with the LUC reporter driven by the 1.6-kb promoter region of *TaCHSL-A2* (Fig. 4C). Co-expression of the *Ta*MYB-D7b effector and LUC reporter in *Nicotiana benthamiana* leaves resulted in a 3-fold increase in LUC reporter activity compared with the control (Fig. 4D). In contrast, co-expression of the *Ta*MYB-D7a effector did not produce a significant effect (Fig. 4D). These findings indicate that the transcription of *TaCHSL-A2* was activated by *Ta*MYB-D7b but not by *Ta*MYB-D7a.

### Gene-editing events and phenotypes of *Ta*MYB7

**Figure 5.**
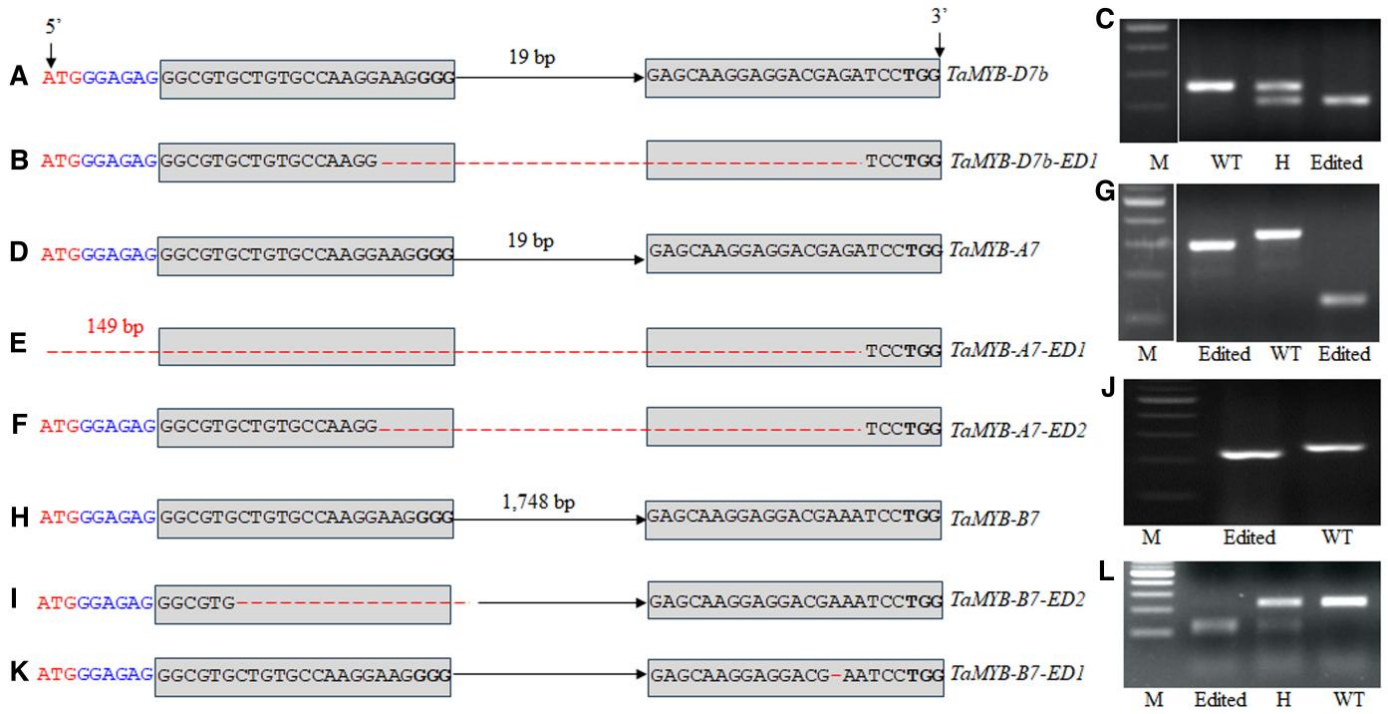
Editing events at *Ta*MYB7. The 2 sgRNAs are shown in the gray rectangles, the PAMs are denoted by boldface type, the ATG (translation start site) is shown in red, and the sequence between the ATG and the first sgRNA is shown in blue. **A)** Wild-type sequence of *Ta*MYB-D7b. **B)** Sequencing of the *Ta*MYB-D7b-ED1 editing event. Deleted bases are shown as red dotted lines. **C)** PCR genotyping for the deletion in *Ta*MYB-D7b-ED1. **D)** Wild-type sequence of *Ta*MYB-A7. **E)** Sequence of the *Ta*MYB-A7-ED1 editing event, showing a 149-bp deletion upstream of the first sgRNA. **F)** Sequence of the *Ta*MYB-A7-ED2 editing event. **G)** PCR genotyping for the deletions in *Ta*MYB-D7b-ED1 and *Ta*MYB-A7-ED2. **H)** Wild-type sequence of *Ta*MYB-B7. **I)** Sequence of the *Ta*MYB-B7-ED1 editing event. **J)** PCR genotyping for the deletion in *Ta*MYB-B7-ED1. **K)** Sequence of the *Ta*MYB-B7-ED2 editing event. **L)** PCR genotyping for the 1-bp deletion in *Ta*MYB-B7-ED2. **M)** DNA marker; H, heterozygous.

To validate the functions of *Ta*MYB7, we opted to edit the *TaMYB7* genes in wheat. We designed 2 single guide RNAs (sgRNAs) to edit *TaMYB7* using the clustered regularly interspaced short palindromic repeats (CRISPR)/CRISPR-associated protein 9 (Cas9) system (Wang et al. 2018). The first sgRNA was 9 bp downstream of the start of the ATG, with the second sgRNA 50 bp downstream from the ATG (Supplementary Fig. S4). The 2 sgRNAs target exon 1, with 19 bp separating the protospacer adjacent motif (PAM) of sgRNA1 and the beginning of sgRNA2 (Supplementary Fig. S4). We suspected the 2 sgRNAs would also target the other homoeologous genes, *TaMYB-A7* and *TaMYB-B7*, as the target sites were conserved. *TaMYB-A7* has the same gene structure as *TaMYB-D7*, whereas *TaMYB-B7* has an additional 1,728-bp intron within exon 1 that separates the 2 sgRNA target sites (Supplementary Fig. S4). We transformed the CRISPR/Cas9 gene-editing construct containing the 2 sgRNAs into cv. 2174 carrying the functional *TaMYB-D7b* allele. We identified 1 T_0_ positive plant, and using this plant, generated T_1_ and identified several editing events in 3 homoeologous *TaMYB7* genes. *TaMYB-D7b* (Fig. 5A) harbored a 42-bp deletion between the 2 sgRNAs; we designated this editing event *TaMYB-D7b-ED1* (Fig. 5B, Supplementary Fig. S5). The deletion was large enough to be tested by PCR with the primers designed to sequence the edited sites (Fig. 5C). Similarly, we identified 2 editing events in *TaMYB-A7: TaMYB-A7-ED1*, and *TaMYB-A7-ED2* (Fig. 5D). *TaMYB-A7-ED1* led to a 208-bp deletion (Fig. 5E, Supplementary Fig. S6), while *TaMYB-A7-ED2* resulted in the equivalent 42-bp deletion as seen in *TaMYB-D7-ED1* (Fig. 5F, Supplementary Fig. S5). Again, the deletions were each large enough to allow PCR genotyping (Fig. 5G). Finally, we identified 2 editing events in *TaMYB-B7*, designated *TaMYB-B7-ED1* and *TaMYB-B7-ED2*. *TaMYB-B7-ED1* (Fig. 5H) showed a deletion of 21 bp (Fig. 5I, Supplementary Fig. S7), which is sufficient for PCR genotyping (Fig. 5J). *TaMYB-B7-ED2* showed a 1-bp deletion within the target site of sgRNA2 (Fig. 5K, Supplementary Fig. S7). We designed a derived cleaved amplified polymorphic sequence (dCAPS) marker for *TaMYB-B7-ED2* that only created an *EcoR*I restriction site in the edited allele (Fig. 5L).

### Consequences of edited *Ta*MYB7 genes in transgenic wheat plants

**Figure 6.**
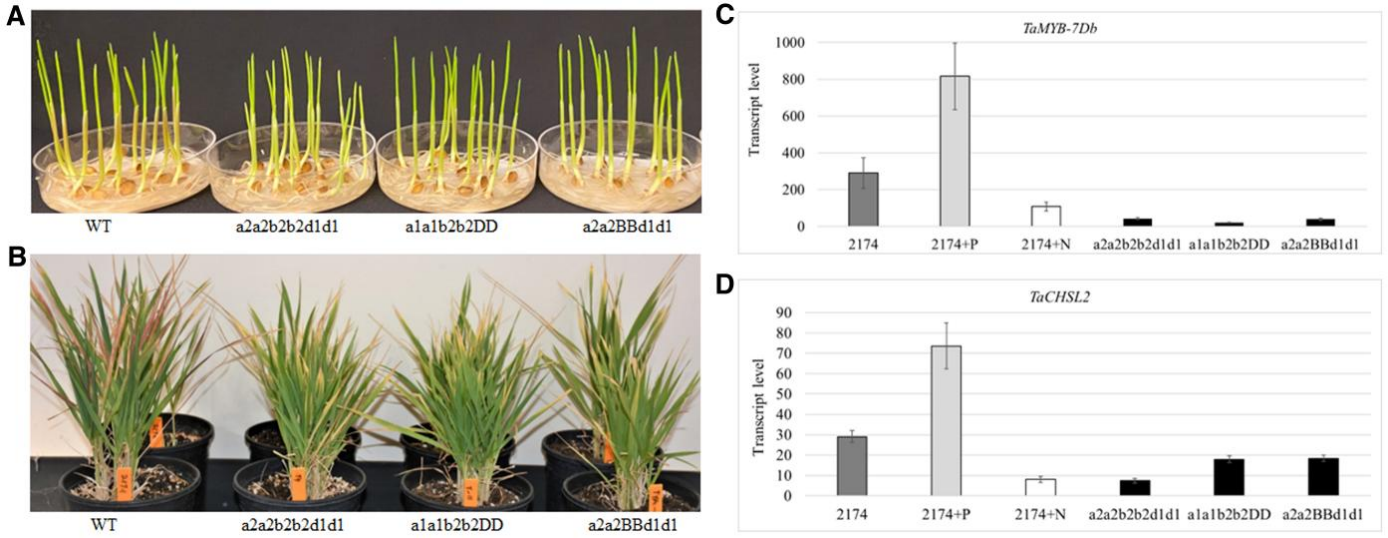
Effects of edited *Ta*MYB7 genes in wheat. **A)** Representative photographs of 2174 seedings (WT) and 2174 seedlings edited for *Ta*MYB7. The seeds were germinated on petri dishes with water and without any nutrients. **B)** Representative photographs of 2174 plants (WT) and 2174 plants edited for *Ta*MYB7. The plants were tested with commercial potting mix without adding any N or P. WT is for wild-type 2174; aa, bb, or dd represent the subgenomes with edited *Ta*MYB7 genes in 2174, a1 for *Ta*MYB-A7-ED1 with a 208-bp deletion, a2 for *Ta*MYB-A7-ED2 with a 42-bp deletion, b2 for *Ta*MYB-B7-ED2 with a 1-bp deletion, and d1 for *Ta*MYB-D7b-ED1 with a 42-bp deletion. AA, BB, or DD represent the wildtypes of *Ta*MYB7 genes in 2174. **C)** Relative *Ta*MYB-D7b transcript levels in 2174 and *Ta*MYB7-edited plants. **D)** Relative *Ta*CHSL2 transcript levels in 2174 and *Ta*MYB7-edited plants. 2174 + P, sample collected 1 wk after P fertilizer was added; 2174 + N, collected 1 wk after N fertilizer was added. Transcript levels were determined by qPCR, with *Actin* as a reference transcript. The gene transcript levels were calculated using the 2^−ΔΔCT^ method, where CT is the threshold cycle. Values are means ± standard error (*n* = 6).

We targeted the sgRNAs at the beginning of the coding sequence to maximize our chances of obtaining strong or null alleles in each gene. We isolated the edited plants with knockouts for 1, 2, or all 3 *TaMYB7* homoeologs, alleviating the need for crosses to stack the edited genes. Transgenic plants harboring one or multiple edited *TaMYB7* homoeologs developed normally and matured. Importantly, the purple color that normally accumulates in the coleoptiles of the parental cv. 2174 disappeared in the plants with *TaMYB7* editing of the homoeologs from the A and B subgenomes, the A and D subgenomes, or all 3 subgenomes (Fig. 6A). When we tested these *TaMYB7*-edited plants on soil lacking N and P, they did not develop a purple color in any tissue, compared with the intense purple color seen in wild-type 2174 plants (Fig. 6B). We conclude that the loss of *TaMYB7* function in cv. 2174 suppresses the purple pigmentation in this background, supporting that *TaMYB7* was associated with the purple coleoptile and leaf phenotypes seen in cv. 2174.

Unexpectedly, we observed that *TaMYB-D7b* transcript levels were downregulated in the *TaMYB-D7b*-edited plants when severely stressed (Fig. 6C). The insertions/deletions occurred in the exons of the edited *TaMYB-D7b* genes and not in the promoters (Fig. 5C). In addition, *TaMYB-D7b* transcript levels were downregulated by N supply but upregulated by P supply (Fig. 6C). When severely stressed, the *Ta*CHSL2 genes were dramatically downregulated in the *Ta*MYB7-edited plants (Fig. 6D). These results suggest that the *TaMYB7* may act upstream of the *TaCHSL2* promoters to activate their transcription. The transcript level of *TaCHSL2* in Jagger was very low, indicating that the loss of *TaMYB7* function alleviated the transcriptional induction of *TaCHSL2* and thus prevented the biosynthesis of purple pigments in coleoptiles and leaves in Jagger (Supplementary Fig. S8). Under different N and P conditions, the transcript levels of *TaCHSL2* genes in 2174 carrying the edited *aabbdd* genes were much higher than those in Jagger, suggesting that *TaCHSL2* was regulated by *TaMYB7* and other genetic factors.

## Discussion

The first *MYB* (myeloblastosis) gene identified from plants was the *P* gene that controls pigmentation in the floral organs of maize (Grotewold et al. 1994). The Arabidopsis genome encodes 125 MYB proteins (Stracke et al. 2001). Common wheat, a hexaploid species, has over 375 *MYB* genes. In this study, we characterized *TaMYB-D7*, corresponding to *TraesCS7D02G166500* in the CS genome. This gene maps closely to the previously reported *Rc-D1* (Khlestkina et al. 2002), *TaC1-D1* (Himi and Taketa 2015), and *TaMYB-D1* (Cao et al. 2010) genes associated with purple pigmentation of wheat coleoptiles. We describe here the 2 *TaMYB-D7* alleles *TaMYB-D7a* and *TaMYB-D7b*. Importantly, the *TaMYB-D7b* allele was responsible for accumulating purple pigments in the leaves of plants experiencing N and P deficiency. For wheat cultivars harboring the *TaMYB-D7b* allele, purple coleoptiles, and leaves can, therefore, be used as a visible marker of nutritional deficiency and mitigate potential losses in yield by enabling wheat farmers to manage as soon as symptoms appear.

**Figure 7.**
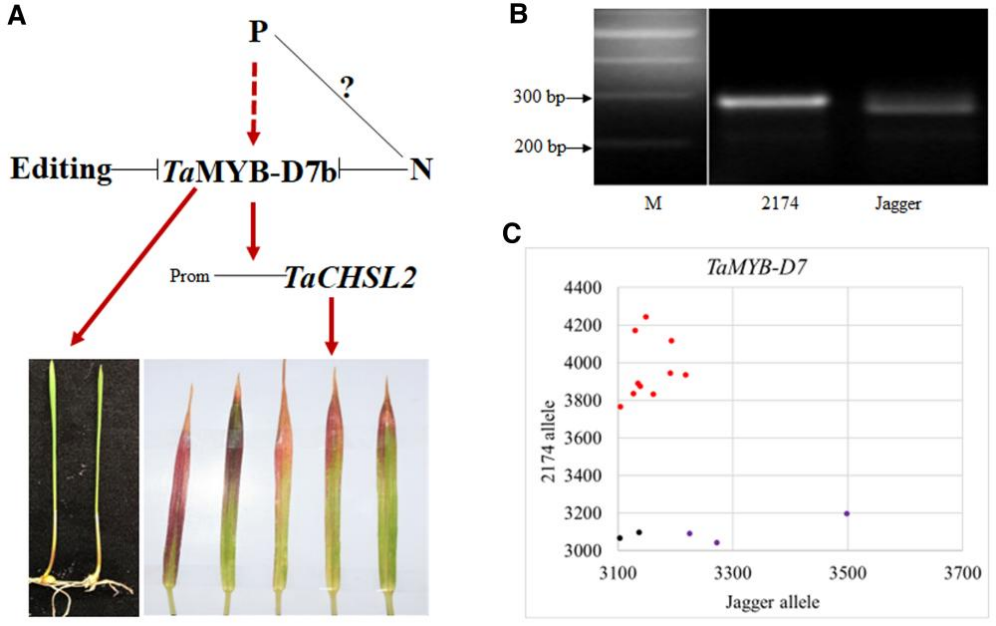
A working model of *Ta*MYB-D7 and its molecular markers. **A)** A working model of *Ta*MYB-D7 controlling purple pigment accumulation in wheat. Arrows indicate promotion, and “-|” indicates repression. P fertilizer induces expression of *Ta*MYB-D7b, whose protein product activates *Ta*CHSL2 transcription. The question mark represents unknown mechanisms in which N and P counteract each other. **B)** PCR marker for the genotyping of the *Ta*MYB-D7 allele in Jagger and 2174. The expected size of the products after digestion with *Pst*I is 273 bp for the 2174 *Ta*MYB-D7b allele and 254 bp + 19 bp (out of the agarose gel) for the Jagger *Ta*MYB-D7a allele. **C)** KASP marker for the genotyping of the allele at *Ta*MYB-D7. KASP-HEX for the 2174 *Ta*MYB-D7b allele is indicated in red, KASP-FAM for the Jagger *Ta*MYB-D7a allele in purple, and the no-template control in black.

We speculate that *Ta*MYB7 may be a transcriptional activator of *TaCHSL2* genes acting in the anthocyanin biosynthetic pathway (Dao et al. 2011). *TaCHSL2* transcript levels were lower in the *TaMYB7*-edited plants, and the edited plants did not accumulate purple pigment in soils lacking N and P, unlike the parental cv. 2174. Notably, *TaMYB7* and *TaCHSL2* transcript levels were both induced by P supply. We propose the following model of the regulatory pathway underlying the purple color in wheat (Fig. 7A). *TaMYB-D7b* is associated with the formation of the purple color in coleoptiles, which is not regulated by the environment or nutrient status, and the appearance of the purple color in leaves, which occurs in response to inadequate N and is exacerbated by low P but can be rescued by supply with sufficient N and P. The natural allele *TaMYB-D7a* (carrying a point mutation in the coding sequence) and knockouts via editing of *TaMYB-D7b* inhibit the formation of purple pigments in coleoptiles and leaves. The functional transcription factor *TaMYB-D7b* may initiate the transcription of *TaCHSL2* to *produce anthocyanidins, leading* to a purple color in wheat tissues. *TaMYB-D7b* transcription was downregulated by N but upregulated by P *via* an unknown mechanism. *TaMYB-D7b* may form a positive feedback loop with its own transcription, as *TaMYB-D7b* transcript levels were significantly downregulated in the *TaMYB-D7b*-edited plants. It is likely that the purple color in coleoptiles and leaves was regulated by different mechanisms. In addition, it remains unclear how the balance between N and P affects the production of purple pigments in the leaves. Leaves turning purple is a standard diagnostic of P deficiency in different plant species (Osborne et al. 2002; Ticconi and Abel 2004; Chen et al. 2014; Flores et al. 2021; Siedliska et al. 2021). In this study, we showed that wheat purple pigments are produced in certain growth phases under diverse environmental conditions. When we tested cv. 2174 plants in 4 wk at the third- or fourth-leaf stage, the first leaves were purple when P or N was inadequate. When we tested cv. 2174 plants in sandy soils at 14 wk (the joining stage), the leaf tips of older leaves were strongly purple under P deficiency and slightly purple in the absence of sufficient N. The purple color of older leaves or in the older part of the same leaf suggests that P is mobile. The mobility of P in plants was reported in different species (Shen et al. 2011). It is worth noting that the leaves of winter wheat may become purple during the winter season in response to low temperatures (Zhu et al. 2013). A comprehensive comparison of the transcript levels of *TaCHSL2* in plants with various edited events in different developmental stages under different N and conditions will be performed in future studies.

The purple coleoptile is an excellent diagnostic indicator of the presence of the *TaMYB-D7b* allele in wheat cultivars. The presence of the *TaMYB-D7b* allele in a wheat cultivar can also help inform farmers on the state of P and N nutrition based on the development of purple leaves. Notably, this visible phenotype is unavailable in other wheat cultivars like Jagger, in which *TaMYB-D7a* is nonfunctional. It is, therefore, critical to ensure that a given wheat cultivar harbors the *TaMYB-D7b* allele before using the absence of purple pigmentation as a sign of nutritional sufficiency or high nutrient use efficiency. Validating the function of the single *TaMYB-D7b* allele is essential. To achieve this, we attempted to cross different edited *TaMYB7* lines with the wild type. The resulting lines are expected to have all 8 genotypes, including those with single and combined edits (*aa, bb, dd, aabb, aadd, bbdd, aabbdd*, and *wild-type*), for future studies. In this study, we developed a simple PCR marker for the SNP distinguishing between *TaMYB-B7a* and *TaMYB-D7b* alleles as a diagnostic and prognostic signature (Fig. 7B). We also created a KASP marker for *TaMYB-B7b* (Fig. 7C), which can be used for high-throughput genotyping in wheat breeding.

## Materials and methods

### Plant materials and soils

A population of RILs was generated using 2 locally adapted winter wheat (*Triticum aestivum L*.) cultivars: “Jagger” and “2174,” which were genotyped using SSR markers. The naked eye evaluated the purple color on a scale of 0 to 10, with 0 for completely green and 10 for completely purple, as previously described (Zhu et al. 2013). The 144 RILs were genotyped using SNP markers on an Illumina Infinium 9 K iSelect platform (Li et al. 2013). The selected SSR and SNP markers were integrated with phenotypes to identify QTLs.

Plants for the Jagger and 2174 cultivars were tested in sand soil, and nutrients were supplied as Hoagland nutrient solution (Supplementary Tables S1 and S2). N or P was supplied at one-sixth of the normal levels in the Hoagland nutrient solution to test the effects of N and P deficiency on wheat leaf color. The sand was washed with tap water every 10 days to avoid the accumulation of salt ions. The plants were maintained in a greenhouse, with a constant temperature of 20 to 25 °C and a 16-h light/8-h dark photoperiod provided for the entire life cycle (Liu et al. 2018a, 2018b).

The cv. 2174 was used as the recipient for transformation with *TaMBY7* gene editing constructs. The resulting T_0_-positive plants and T_1_ populations were genotyped. Seeds from *TaMBY7* gene-edited plants and the parental line 2174 were germinated in Petri plates with paper towels soaked with water, and the seedlings were transplanted at the one-leaf stage into pots filled with commercial potting mix (Lei et al. 2018). All plants (edited families and the wild-type controls) were grown without fertilization in the same greenhouse.

### Transcript levels of genes

Samples were collected from the roots and shoots of 5 seedlings at the 5- and 6-leaf stages grown in commercial soil. Total RNA was extracted from the harvested leaves using TRIZOL reagent (INVITROGEN) and reverse-transcribed to first-strand cDNA using a poly(dT) primer. End-point qPCR was performed to determine the transcript levels of *TaMYB-D7* and *TaCHSL2* using a SYBR Green I kit (Bio-Rad). The forward primer *Ta*MYB-D7-RT-F2 and the reverse primer *Ta*MYB-D7-RT-R2, both in exon 2, were designed for *Ta*MYB-D7. The resulting PCR product is 194 bp. The forward primer CHS-RT-F1, which is in exon 1, and the reverse primer CHS-RT-R1, where the underlined 6 bp are from exon 1, and the remaining 16 bp are from exon 2, were designed for *TCHSL2*. The PCR product is 192 bp. Each qPCR consisted of an initial denaturation of 2 min at 95 °C, followed by 40 cycles of 95 °C for 15 s, 55 °C for 20 s, and 72 °C for 31 s.

### Editing of *Ta*MYB7

Two single guide RNAs (sgRNAs) were designed to target *TaMYB7*: *TaMYB7*-CRISPR-F (5′-GGCGTGCTGTGCCAAGGAAGGGG-3′) and *TaMYB7*-CRISPR-F (5′-GAGCAAGGAGGACGAGATCCTGG-3′) with the underlined sequences denoting the PAM (Jia et al. 2021). The 2 sgRNAs target 2 sites that are separated by 19 bp. The first sgRNA was 9 bp downstream from the beginning of the ATG start codon to introduce early termination of translation. The designed sgRNAs were expected to target all 3 homoeologs within each common wheat sub-genome (A, B, and D). The sgRNA sequences were used to search the IWGSC RefSeq v2.1 CS genome sequence using BLAST to ensure no potential off-target insertion sites. Each sgRNA sequence and its complementary sequences were cloned into the pBUN421 vector, which harbors the *Cas9* gene optimized for maize (*Zea mays*) codon usage (Wang et al. 2018). The resulting constructs were co-transformed into cv. 2174 embryos using biolistic bombardment with DNA-coated gold particles. Positive T_0_ plants with the editing constructs inserted in the wheat genome were allowed to self-pollinate to generate T_1_ populations.

Specific primers were designed to amplify the genomic region covering the sgRNA target sites in *TaMYB1* from the positive plants. *Ta*MYB-A7-ED-F1 and *Ta*MYB-A7-ED-R1 were used to amplify the *TaMYB-A7* target region. *Ta*MYB-B7-ED-F1 and *Ta*MYB-B7-ED-R1 were used to amplify the *TaMYB-B7* target region. *Ta*MYB-D7-ED-F1 and *Ta*MYB-D7-ED-R1 were used to amplify the *TaMYB-D7* target region. The PCR products were sequenced directly by Sanger sequencing. PCR products of unexpected size were also Sanger sequenced directly. Chromatograms with sharp peaks with very little noise were analyzed to determine the nature of the editing event, presumed to be homozygous in the corresponding plants. When overlapping peaks were observed around the sgRNA target sites, the PCR products were cloned into the pGEM vector for Sanger sequencing of individual clones, with the assumption that these plants are bi-allelic.

### Protein expression and EMSA

For the expression of His-tagged proteins, truncated coding regions of *TaMYB-7Da* and *TaMYB-7Db* (corresponding to amino acids 1 to 119) were amplified and cloned into the pET32a vector (Merck Millipore, USA). The resulting plasmids were transformed into *Escherichia coli* strain BL21 (DE3) and induced using 0.8 mm isopropyl β-D-thiogalactopyranoside (IPTG) for 6 h at 25°C. His-tagged *Ta*MYB-7Da and *Ta*MYB-7Db proteins were purified using Ni-NTA resin (Qiagen, Germany).

The EMSA was performed as described previously (Jian et al. 2022). Briefly, 5′-biotin-labeled primers were synthesized, annealed, and used as probes. The biotin-labeled probes (100 fmol) were incubated with 1 *µ*g His-*Ta*MYB-7 Da or His-*Ta*MYB-7Db protein in a 20 *µ*L reaction mixture comprising 1× binding buffer, 2.5% glycerol, 5 mm MgCl_2_, and 1 *µ*g poly (dI-dC) for 30 min at room temperature. Protein-DNA complexes were detected using a Light Shift Chemiluminescent EMSA Kit (Thermo Scientific, Rockford, IL, USA) according to the manufacturer's instructions.

### Transient assays in *Nicotiana benthamiana* leaves

Transient assays of transcriptional activity were conducted in *N. benthamiana* leaves using a dual-luciferase reporter system as described (Hellens *et al.* 2005). In brief, a 1.6-kb promoter sequence of *TaCHSL-A2* was cloned into the pGreenII 0800-LUC reporter plasmid. The full-length coding regions of *TaMYB-7Da* and *TaMYB-7Db* were cloned into the 1300-GFP effector plasmid. The resulting reporter and effector constructs were transformed into *Agrobacterium tumefaciens* strain GV3101 cells. These cells were then co-infiltrated into *N. benthamiana* leaves. After 72 h incubation, relative LUC activity (LUC/REN) was measured using the Dual-Luciferase Reporter Assay System (Promega, Madison, WI, USA).

### Molecular markers for the natural *Ta*MYB-*7d* alleles

Two allele-specific primers were designed, *Ta*MYB-D7-dCAP-F1 and *Ta*MYB-D7-dCAP-R1. PCR was performed using LongAmp *Taq* DNA polymerase (New England BioLabs) and 35 cycles consisting of 93 °C for 30 s, 60 °C for 30 s, and 72 °C for 30 s, after an initial denaturation at 95 °C for 5 min. The PCR products were directly digested with the restriction enzyme *Pst*I. Following digestion, the PCR products were separated on a 2% (w/v) agarose gel for genotyping, with the Jagger *TaMYB-D7a* allele product in a 254-bp and a 19-bp band, while the 2174 *TaMYB-D7b* allele resolves as a single 273-bp band.

A Kompetitive Allele-Specific PCR (KASP) marker was also developed for high-throughput genotyping of the allele at *TaMYB-7D*. A specific primer, *Ta*MYB-D7-KASP-F1, was designed to bind to both alleles of *TaMYB-D7*. Two allele-specific primers were also designed: *Ta*MYB-D7-KASP-R1 for the Jagger allele and *Ta*MYB-D7-KASP-R2 for the 2174 allele. The total amplicon length is 148 bp. PCR conditions consisted of a hot start at 94 °C for 15 min, followed by 10 touchdown cycles (94 °C for 20 s; touchdown at 65 to 57 °C initially and decreasing by 0.8 °C per cycle for 60 s), followed by 30 additional cycles (94 °C for 20 s; 57 °C for 60 s). Fluorescence intensities were measured on an ABI-7500 Real-time PCR system following the manufacturer's guidelines to run KASP reactions.

All primers used in this study are provided in Supplementary Table S3.

### Accession numbers

*Ta*MYB-D7c sequence data from this article can be found in the GenBank/EMBL data libraries under accession number MG066454.

## Supplementary Material

kiaf224_Supplementary_Data

## Data Availability

The data supporting this study's findings are available from the corresponding author upon reasonable request.

## Supplementary data

The following materials are available in the online version of this article.

**Supplementary Figure S1.** The sequences of gDNA of *TaMYB-D7* gene in different cultivars.

**Supplementary Figure S2.** Sequences of MYB7 proteins.

**Supplementary Figure S3.** The promoter sequences of three homoeologous *TaCHSL2* genes.

**Supplementary Figure S4.** The sequences and locations of two sgRNAs in the *TaMYB7* genes.

**Supplementary Figure S5.** The chromatogram sequence of *Ta*MYB-D7b-ED1.

**Supplementary Figure S6.** The chromatogram sequence of *TaMYB-A7* edited alleles.

**Supplementary Figure S7.** The chromatogram sequence of *TaMYB-B7* edited alleles.

**Supplementary Figure S8.** The transcript levels of *TaCHSL2* genes in natural and edited mutants of *TaMYB7*.

**Supplementary Table S1.** N or P content in different treatments in sand culture.

**Supplementary Table S2.** Other macronutrients in sand culture.

**Supplementary Table S3.** Primers used in this study.

## Dive Curated Terms

The following phenotypic, genotypic, and functional terms are of significance to the work described in this paper:

MYB7 Gramene: AT2G16720
MYB7 Araport: AT2G16720

MYB63 Gramene: AT1G79180
MYB63 Araport: AT1G79180
