# Supplementary material for: A natural allele of the transcription factor gene TaMYB-D7b is a genetic signature for phosphorus deficiency in wheat
Source: Plant Physiol. 2025 Jul 16;199(3):kiaf224. doi: 10.1093/plphys/kiaf224 (PMC12637205; doi:10.1093/plphys/kiaf224)
Supplement: kiaf224_Supplementary_Data [file kiaf224_supplementary_data.pdf]

Supporting Information

A natural allele of the transcription factor gene *TaMYB-D7b* is a genetic signature for deficiency of phosphorus and nitrogen in wheat

Daijing Zhang, Min Fan, Tian Li, Yahya Rauf, Yongjie Liu, Xinkai Zhu, Haiyan Jia, Wenxuan Zhai, Juan C. Luzuriaga, Brett F. Carver, Liuling Yan*

The following Supporting Information is available for this article:

Supplementary Figure S1 The sequences of gDNA of *TaMYB-D7* gene in different cultivars.

Supplementary Figure S2 Sequences of MYB7 proteins.

Supplementary Figure S3 The promoter sequences of three homoeologous *TaCHSL2* genes.

Supplementary Figure S4 The sequences and locations of two sgRNAs in the *TaMYB7* genes.

Supplementary Figure S5 The chromatogram sequence of *TaMYB-D7b-ED1*.

Supplementary Figure S6 The chromatogram sequence of *TaMYB-A7* edited alleles.

Supplementary Figure S7 The chromatogram sequence of *TaMYB-B7* edited alleles.

Supplementary Figure S8 The transcript levels of *TaCHSL2* genes in natural and edited mutants of *TaMYB7*.

Supplementary Table S1 N or P content in different treatments in sand culture.

Supplementary Table S2 Other macronutrients in sand culture.

Supplementary Table S3 Primers used in this study.

```
>TraesCS7D02G166500
ATGGGGAGGAGGGCGTGCTGTGCCAAGGAAGGGGTGAAGCGAGGGGCCTGGACGAGCAAGGAGGACGAGATCCTGGCTTCCTACGTCAAGGCCCATG
GCGAAGGCAGGTGGAGGGAGCTCCCGCAGCGAGCTGGTACTACTCGCGAGATGTTAGCTACCGATCCATCGTAAACCGTTCTGGTTCTATCCAATGG
AGAGCTGGAATGGAAGTGACGCATGCTGCTAACTGAATGAATCTGCATGCAGGTCTGCGGCGGTGCGGCAAGAGCTGTTGGAATAAGCCACGGCGTG
TGTGGTCGGGTTCGTCGGGCGCGTCGGGTGCGCGCGGGACGGGCGGGACATACTGGTGCCGTCGGGCTCGTCGGGTTTGTCGGGCGTGTCGGTGACG
CGCGGGACGGACGGGACGTGCGGGACGCAGCAGCGTGGCGTGTGTGTGTATGGGCAGTGTATGGCAAAGTGTGCGTGCGTGTGTGACGTGGCGGTGT
GAGTTAGCTAGCTAGACGGGCGTGCATGCATGAATTAGTTGCTGTGTAGTAGTCTGGCTGTTAGCAGCGGCGGCCGCGTCGTGCGCGAAGACCGAGT
GGATCGCCTGCATGTAGTTGGGCACGTTCGTGCATGCGTAGGAAGCGTAGGCGTTGCGTTGCTGCTGGGCTACAAAGCCAGCCGGTTTGTATGTGTT
TGAAATGAGCTAGTGTAGTGAGAAGAAAAGAAAAGACGCCGGGGCACTGAACGCCGGGGCGTTGGTCACTAGAAAGTGTTTCTTTCTTCTACCTCCA
TCTATCCGTGTGAGAGAGCTGCAACAACAATTGGTATACAGAGCTTCGGTTCGGGCGATCACCGGCGACCATGGCGCTCGTCCCACACGGCGGTGGC
GCGGGCGGATCGGCGACGATGGCGATGCCGATGCTGACCGCGGACAACTACACGGTCTGGGCCATCAAGGCGCAGGCGATCCTCGACGTCCACACCG
TATGGGAGGCGGTGGCGCCGGGCGACGCGGCGGTGAACGCGCGGAAGGACAAGATGGCGCGTGCGTTGCTTCTCGGGGCGTTGCCGGAGGATGTGCT
GCTGCAGGTGTCGACGAAGCTCACCGCCAGGGAGGTATGGGACTCCCTGAAGGTGAGGTTCGTCGGCGCCGATCGGGTCCGTGCGGCGAGGCTGGGG
ACGCTGCGCGGCGAATTCGACCGGATGAAGATGGCGGACGGCGAGGAGCTCGATGTGTACGGCGGGAGGCTCGCGGCGATGGCGGCGAGGTATGCCA
ACCTCGGGGAGACGCTGGGCGACGCAGCACTTGTCAAGAAGTTGCTGGATACGGTGCCGGATCGCCTCTTCCCCGTCGTCGCCGGCATCGAGCAGTT
CCACGACGTGACGACGATGGCGTTCGACGAAGCGCTCGGGCGGCTGCGTGCGTTCGACGAGCGGGTTCGGCGCCGTGGACAAGACGGTGGCGAGCGC
GGGGGTGAGCAGCTGCTCATGACGGCGGCGCAGTGGGCAGCGCGGGAGCGTCGACACGGCGGTGCTCGGGACGACGACGACGGGCGCAGCGTGGCGT
CGGGGAGCGGCGGCAACAGGCGCAGGCGCTGCTACAAGTGCGGGGAGCACGGGCACTTCCGGCGCGAGTGCCCGCAGCTGCGGAAAGGACCGGCGGC
GGAGCAGGCTCTCTTGGCCGGTGCCAACGTTGACGACGACGGACTCCTCTAGGCCGTGGCTTAGGGGGTGTGTGTTGGAATAAGCCACGGCGTGTGT
GGTCGGGTTCGTCGGGCGCGTCGGGTGCGCGCGGGACGGGCGGGACATACTGGTGCCGTCGGGCTCGTCGGGTTTGTCGGGCGTGTCGGTGACGCGC
GGGACGGACGGGACGTGCGGGACGCAGCAGCGTGGCGTGTGTGTGTATGGGCAGTGTATGGCAAAGTGTGCGTGCGTGTGTGACGTGGCGGTGTGAG
TTAGCTAGCTAGACGGGCGTGCATGCATGAATTAGTTGCTGTGTAGTAGTCTGGCTGTTAGCAGCGGCGGCCGCGTCGTGCGCGAAGACCGAGTGGA
TCGCCTGCATGTAGTTGGGCACGTTCGTGCATGCGTAGGAAGCGTAGGCGTTGCGTTGCTGCTGGGCTACAAAGCCAGCCGGTTTGTATGTGTTTGA
AATGAGCTAGTGTAGTGAGAAGAAAAGAAAAGACGCCGGGGCACTGAACGCCGGGGCGTTGGTCACTAGAAAGTGTTTCTTTCTTCTACCTCCATCT
ATCCGTGTGAGAGAGCTGCAACAACAAGAGCTGCCGGCTGCGGTGGCTGAACTACCTCCGGCCGAACATCAAGCGGGGCAACATCTCCGACGACGAG
GAGGAGCTCATCGTCAGGCTCCACGGCCTGCTCGGCAACAGGTGGTCCATCATCGCCGGCAGGCTGCCCGGCCGAACAGACAACGAAATCAAGAACT
ACTGGAACAGCACGCTCGGCAGGAAGGCGCTCCCCGCCCGCCCCGCCATTGCCGCGGCGAGGACCGTCGCCTCCGGCTCCTCCAGCTCCACGGGGAG
CGCCGCGGCGGCGCTGTCTACCTCCGTCCCTGCCGTCCTCCATGGCGCGGCGCCTTCGTCGCCGGCCGGCGCGGTGTGGGCGCCCAAGCCCGTGAGG
TGCACGGGCGGCCTCTTCTTCCGCAGGGAGCAGGAGACGCCGCCGCCCGCGCCGGTCGTCGAGGAGACGCGGGCCGGAGGAGATCAAGAGGGAGACG
CCTGCAGCGGCAGCAGCTCGGAGACGTCGTCGGCCGAGCCGTGCTCGTCGGGGTCGGGCGGCGGAGACTGGATGGACGACGTGAGAGCCCTGGCGTC
GTTCCTGGAGTCCGACGAGGAATGGCTCAAGTCCCTGCACATGGCCGGTTAATTAACCTCTCACACGTGATCGATCTGGTTCAGTCACATGTACGTG
GGTGCGCCGCTGCACGTACGCTTTTCGAAAAGGAAACTGTATGTATGCTTGAGCAGTGTGCATACGTACAAACGTACTTACGCCCGCGCGTATGTGG
CGGTACGTGGCTGGCTA
>TaMYB-D7a-Jagger
ATGGGGAGGAGGGCGTGCTGTGCCAAGGAAGGGGTGAAGCGAGGGGCCTGGACGAGCAAGGAGGACGAGATCCTGGCTTCCTACGTCAAGGCCCATG
GCGAAGGCAGGTGGAGGGAGCTCCCGCAGCGAGCTGGTACTACTCGCGAGATGTTATCATGTTAGCTACCGATCCATCGTAAACCGTTCTGGTTCTA
TCCAATGGAGAGCTGGAATGGAAGTGACGCATGCTGCTAACTGAATGAATCTGCATGCAGGTCTGCGGCGGTGCAGCAAGAGCTGCCGGCTGCGGTG
GCTGAACTACCTCCGGCCGAACATCAAGCGGGGCAACATCTCCGACGACGAGGAGGAGCTCATCGTCAGGCTCCACGGCCTGCTCGGCAACAGGTGG
TCCATCATCGCCGGCAGGCTGCCCGGCCGAACAGACAACGAAATCAAGAACTACTGGAACAGCACGCTCGGCAGGAAGGCGCTCCCCGCCCGCCCCG
CCATTGCCGCGGCGAGGACCGTCGCCTCCGGCTCCTCCAGCTCCACGGGGAGCGCCGCGGCGGCGCTGTCTACCTCCGTCCCTGCCGTCCTCCATGG
CGCGGCGCCTTCGTCGCCGGCCGGCGCGGTGTGGGCGCCCAAGCCCGTGAGGTGCACGGGCGGCCTCTTCTTCCGCAGGGAGCAGGAGACGCCGCCG
CCCGCGCCGGTCGTCGAGGAGACGCGGGCCGGAGGAGATCAAGAGGGAGACGCCTGCAGCGGCAGCAGCTCGGAGACGTCGTCGGCCGAGCCGTGCT
CGTCGGGGTCGGGCGGCGGAGACTGGATGGACGACGTGAGAGCCCTGGCGTCGTTCCTGGAGTCCGACGAGGAATGGCTCAAGTCCCTGCACATGGC
CGGTTAA
>TaMYB-D7b-2174
ATGGGGAGGAGGGCGTGCTGTGCCAAGGAAGGGGTGAAGCGAGGGGCCTGGACGAGCAAGGAGGACGAGATCCTGGCTTCCTACGTCAAGGCCCATG
GCGAAGGCAGGTGGAGGGAGCTCCCGCAGCGAGCTGGTACTACTCGCGAGATGTTATCATGTTAGCTACCGATCCATCGTAAACCGTTCTGGTTCTA
TCCAATGGAGAGCTGGAATGGAAGTGACGCATGCTGCTAACTGAATGAATCTGCATGCAGGTCTGCGGCGGTGCGGCAAGAGCTGCCGGCTGCGGTG
GCTGAACTACCTCCGGCCGAACATCAAGCGGGGCAACATCTCCGACGACGAGGAGGAGCTCATCGTCAGGCTCCACGGCCTGCTCGGCAACAGGTGG
TCCATCATCGCCGGCAGGCTGCCCGGCCGAACAGACAACGAAATCAAGAACTACTGGAACAGCACGCTCGGCAGGAAGGCGCTCCCCGCCCGCCCCG
CCATTGCCGCGGCGAGGACCGTCGCCTCCGGCTCCTCCAGCTCCACGGGGAGCGCCGCGGCGGCGCTGTCTACCTCCGTCCCTGCCGTCCTCCATGG
CGCGGCGCCTTCGTCGCCGGCCGGCGCGGTGTGGGCGCCCAAGCCCGTGAGGTGCACGGGCGGCCTCTTCTTCCGCAGGGAGCAGGAGACGCCGCCG
CCCGCGCCGGTCGTCGAGGAGACGCGGGCCGGAGGAGATCAAGAGGGAGACGCCTGCAGCGGCAGCAGCTCGGAGACGTCGTCGGCCGAGCCGTGCT
CGTCGGGGTCGGGCGGCGGAGACTGGATGGACGACGTGAGAGCCCTGGCGTCGTTCCTGGAGTCCGACGAGGAATGGCTCAAGTCCCTGCACATGGC
CGGTTAA
```

Supplementary Figure S1 The sequences of gDNA of *TaMYB-D7* gene in different cultivars. The start codon, the stop codon, and the splicing sites of the intron are highlighted in yellow and red letters. Exon sequences are highlighted in grey. The exon sequences are highlighted in grey. TraesCS7D02G166500 is a gene from the Chinese Spring. *TaMYB-D7a* in Jagger and *TaMYB-D7b* in 2174 are allelic to TraesCS7D02G166500. Apparently, exon 1 in TraesCS7D02G166500 was split into exons due to an insertion of an intron. The intron was caused by two repeated fragments CAAGAGCTG that are underlined. Only SNP between *TaMYB-D7a* and *TaMYB-D7b* are highlighted in red.

```
>TaMYB-D7-Jagger
MGRRACCAKEGVKRGAWTSKEDEILASYVKAHGEGRWRELPQRAGLRRCSKSCRLRWLNYLRPNIKRGNISDDEEELIVRLHGLLGNRWSIIAGRLP
GRTDNEIKNYWNSTLGRKALPARPAIAAARTVASGSSSSTGSAAAALSTSVPAVLHGAAPSSPAGAVWAPKPVRCTGGLFFRREQETPPPAPVVEET
RAGGDQEGDACSGSSSETSSAEPCSSGSGGGDWMDDVRALASFLESDEEWLKSLHMAG
>TaMYB-D7-2174
MGRRACCAKEGVKRGAWTSKEDEILASYVKAHGEGRWRELPQRAGLRRCGKSCRLRWLNYLRPNIKRGNISDDEEELIVRLHGLLGNRWSIIAGRLP
GRTDNEIKNYWNSTLGRKALPARPAIAAARTVASGSSSSTGSAAAALSTSVPAVLHGAAPSSPAGAVWAPKPVRCTGGLFFRREQETPPPAPVVEET
RAGGDQEGDACSGSSSETSSAEPCSSGSGGGDWMDDVRALASFLESDEEWLKSLHMAG
>TraesCS7A02G165700
MGRRACCAKEGVKRGAWTSKEDEILASYVKAHGEGRWRELPQRAGLRRCGKSCRLRWLNYLRPNIKRGNISDDEEELIVRLHGLLGNRWSIIAGRLP
GRTDNEIKNYWNSTLGRKALPARPTIAAARTVVATPVASGSSSSTGSAAAPLSTSVPALLHAAAPSSPAAAVWAPKPVRCTGGLFFRREQETPPPAP
VVEETRAGGEEGDACSGSSSSETSSAEPCSSGSGGGDWMDDVRAWRHSSSPTRNGSSPCTWPVKTSHT
>TraesCS7B02G070400
MGRRACCAKEGVKRGAWTSKEDEILASYVKAHGEGRWRELPQRAGLRRCGKSCRLRWLNYLRPNIKRGNISDDEEELIVRLHGLLGNRWSIIAGRLP
GRTDNEIKNYWNSTLGRKALPARPAIAAARTIATPVASGSSSSTAGNAAVALSTSVPAVLHAAAPSSPTAAVWAPKPVRCTGGLFFRRETPPPAPVV
AETRAGGEEGDACSGSCSETSSAEPCSSGSGGGDWMDDVRALASFLESDEEWLKSLHMAG
>TraesCS7D02G166500
MGRRACCAKEGVKRGAWTSKEDEILASYVKAHGEGRWRELPQRAGLRRCGKSCRLRWLNYLRPNIKRGNISDDEEELIVRLHGLLGNRWSIIAGRLP
GRTDNEIKNYWNSTLGRKALPARPAIAAARTVASGSSSSTGSAAAALSTSVPAVLHGAAPSSPAGAVWAPKPVRCTGGLFFRREQETPPPAPVVEET
RAGGDQEGDACSGSSSETSSAEPCSSGSGGGDWMDDVRALASFLES
>OAP08362.1 MYB7 [Arabidopsis thaliana]
MGRSPCCEKEHMNKGAWTKEEDERLVSYIKSHGEGCWRSLPRAAGLLRCGKSCRLRWINYLRPDLKRGNFTHDEDELIIKLHSLLGNKWSLIAARLP
GRTDNEIKNYWNTHIKRKLLSKGIDPATHRGINEAKISDLKKTKDQIVKDVSFVTKFEETDKSGDQKQNKYIRNGLVCKEERVVVEEKIGLDLNLEL
RISPPWQNQREISTCTASRFYMENDMECSSETVKCQTEDTSSISYSSIDISSSNVGYDFLGLKTRILDFRSLEMK
>SANT/MYB DBD ID1000535
CCTKMGMKRGPWTVEEDEILVSFIKKEGEGRWRSLPKRAGLLRCGKSCRLRWMNYLRPSVKRGGITSDEEDLILRLHRLLGNRWSLIAGRIPGRTDN
EIKNYWNTHLRKK
```

Supplementary Figure S2 Sequences of MYB7 proteins. The proteins from deduced *TaMYB-D7a* in Jagger and *TaMYB-D7b* in 2174 are compared with the proteins deduced from three homoeologous genes TraesCS7A01G165700, TraesCS7B01G070400 and TraesCS7D01G166500 in Chinese Spring. The protein that has the highest identity in Arabidopsis to these wheat MYB7 proteins is AtMYB7. Only point mutation between *Ta*MYB-D7a and *Ta*MYB-D7b is highlighted in red. SANT/MYB (ID1000535) has the same DNA binding domain (DBD) as the MYB7 proteins.

```
>TraesCS2A02G527700
ATTCCCAAACCTACACGGACGAGCACGTGGAAGTGGGAAGGCCCAAGGCCAAGCAACCCAACTAACTCCCACTTGGGGCGGATTGGTTGGTAGCAAT
GGTAGGTAGACGTGTCGCTCGCTCCCATCTCCTATCCGCGCATTCGTGGCCATCCATCCTCCCGTCCAATGGACCTAACCCCGTCCTCCCAACGGCC
AGCTTTCCCTTACTTACCCACCCTTCCCGCCTATATATCCCGTCGCCCGCACTCTCATGGCACCACAACACACCACGCCAGTACAAGCGGCGCCTGT
CATTTGTCTACCACTCTGCTAGCTGCTTCCCTCTGCGTCCGGCGAACTTAGACGTCACACAGCCGGCCACTGGTGCGACAACTCCTAGCTCGTCGGC
CGCTGGTACGTGCCTACGTAGATCCATCGATG
>TraesCS2B02G558400
ATTCCCAAACCTACACAGACGAGCACGTGCAACTGGGAAGGCCCAAGGCCAAGCAACCCAACTAACCCCCACTTGGGGCGGATTGGTTGGTAGCAAT
GGTAGGTAGACGTGTCGCTCGGCCCTCATCTCCCTATCCCCGCATTCGTGGCCATCCATCCTCCCGTCCGATGGACCTAACCCCCTTCTCCCAACGG
CCAGCTTTCGCTTACTTACCCACCCTTCCCGCCTATATATCCCGTCGCCCATACTCTCATGGCACCACACCACACCACGCCACCAGTACAAGCGGCG
CCTGTCATTTGTCTACCACCTGCTAGCTAGCTGCTTCCCTCTGCGCCCCGCAAACTTAGACTTCACACAGCCGGCCACTGGTGCGACAACTACTAGC
TCGTCGGCCGCTGGTACGTACGTAGATCCCATCGATG
>TraesCS2D02G530600
AATTCCCAAACCTACACAGACGAGCACGTGCAAGTGGGAAGGCCCAAGGCCAAGCAACCCAACTAACTCCCACTTGGGGCGGATTGGTTGGTAGCAA
TGGTAGGTAGACGTGTCGCTCGGCCCTCATCTCCTGCCCGCGCATTCGTGGCCATCCATCCTCCCGTCCAATGGACCTAACCCCCTTCTCCCAACGG
CCAGCTTTCCCTTACTTACCCACCCTTCCCGCCTATATATCCCGTCGCCCATACTCATGGCACCACACCACACCAGTACAAGCAGCGCCTGTCATTT
GTCTACCACCTGCTAGCTGCTTCCCTCTGCGTCCCGCAAACTTAGACGTCACGCAGCCGGCCACTGGTGCGACAACTCCTAGCTCGTCGGCCGCTGG
TACGTACGTAGATCCATCGATG
```

Supplementary Figure S3 The promoter sequences of three homoeologous *TaCHSL2* genes. The start codon is highlighted in yellow and red letters. The potential DNA binding motifs, ACCTAC, GGATGGT, GGTAGGT, CCTACC, and ACCTAAC, are highlighted in different colors. These DNA binding motifs may be targets of transcriptional factors *Ta*MYB7.

>TraesCS7A02G165700
ATGGGAGGAGGGCGTGCTGTGCCAAGGAAGGGGTGAAGCGAGGGGCCTGGACGAGCAAGGAGGACGAGATCCTGGCTTCCTACGTCAAGGCCCATG
GCGAGGGCAGGTGGAGGGAGCTCCCCCAGAGAGCTGGTACGTACTGATCCATCGTAAACGCATTCTGGTTCTATCAATGGGGAGCTGGAATGGAAGT
GACGCATGCTAACTGAATGAATCTGCATGCAGGTCTGCGGCGGTGCGGCAAGAGCTGCCGGCTGCGGTGGCTGAACTACCTCCGGCCGAACATCAAG
CGGGGCAACATCTCCGACGACGAGGAGGAGCTCATCGTCAGGCTCCACGGCCTGCTTGGCAATAGGTGGTCCATCATCGCCGGCAGGCTGCCCGGCC
GAACAGATAACGAAATCAAGAACTACTGGAACAGCACGCTCGGCAGGAAGGCGCTCCCCGCCCGCCCCACCATTGCCGCGGCGAGGACCGTCGTCGC
CACGCCCGTCGCCTCCGGCTCCTCCAGCTCCACGGGGAGCGCCGCGGCGCCGCTGTCCACCTCCGTCCCTGCCCTCCTCCACGCCGCGGCACCTTCG
TCGCCGGCCGCCGCGGTGTGGGCGCCCAAGCCCGTGAGGTGCACGGGCGGCCTCTTCTTCCGCCGGGAGCAGGAGACGCCGCCGCCCGCGCCGGTCG
TAGAGGAGACGCGGGCCGGGGGAGAAGAGGGAGATGCCTGCAGCGGCAGCAGCAGCTCGGAGACGTCGTCGGCCGAGCCGTGCTCGTCGGGGTCGGG
CGGGGGAGACTGGATGGACGACGTGAGAGCCTGGCGTCATTCCTCGAGTCCGACGAGGAATGGCTCAAGTCCCTGCACATGGCCGGTTAAAACCTCT
CACACGTGA
>TraesCS7B02G070400
ATGGGAGGAGGGCGTGCTGTGCCAAGGAAGGGGTGAAGCGAGGGGCGTGGACGAGGGACGCGGATTTTTCCTAACTAGTTAGGCGCGCAACGCGTA
TCTAACCCACACATCCACCAGGAAGTCAAATCAATGAGGCCCACAAGCTGTCAGCTTTGCATGGTATTACAACTACCAACGAATTTCGATAGTGCGT
ACGTGGAGATGTATTCCACCTGATTGGACCGGCTGCGGGGCCAGCCCTATAGATTCGACTGTGGTCTACAGGTGCACATGCATCCTCCCAGGGCCCT
GCTCCCAGTACCACTTTTGTCCTTACCGATTAGTAGTACTGATCTAATAGCTGCCCATGCATGCACGAGCTCGCAGGATGAGTGAAGTGAGTGAACT
CGATTAGATTCCTTAAATGTGCAGATTTTCTTCACTAGTATTAAATGAATTACTGTCTTGGTATAAAACTCTGGTGCTAAAAAGTTCAGTTAAGTTG
CGAACAAACATTCCGAGATAGGGAAACAAAAGTGTTGCTGAAGAATTTCTGAGCCAACAAAAGTGTGGCTGAAGAATTTTTGAAGCTAAATTATTCA
TAGTTTTTGTATTAACATATTATGTAAATATACATTAATTTACTTAGTTGATTTTTTAATGGCGAGCTCTTCAAAAAATAGTCCGCAAAATCCTTCA
TGCTTGTACACAAACGCACCGGGTATGCTAGATTAAAGTTCATTTAAGTGGGCTTTCAGTAAAGTCCTTCGTGTTTGAACACAAAAGCACCGGGCAT
GCTATATTAAAATTTGTCATGCAGGGATCTATCCTTGTATAATCGCCCAATGAATTATGTAAAGTCCTTCATGTAGGTACACAAATGCACCGGGTAT
GCTAGATTAAAGTTTATTTAAGTGGGGTCTATCTTTGTGTCCGGCGCTTTCAGTAAAGTCCTTCGTGTTTGAACACGAAAGCACCGGGCATGCTATA
TTAAAGTTTATCATGCGGGATCTATCCTTGTGCAATCGCCCAGTGAATTACGTAAAGTTCTTCATGCTTGTACAGGAACACACCGGGTATGCTGGAT
TAAAGCGCATTAAGTGGGGGCCTATTTTTGTGCAATCGCACGGTCCTTTCAGTAAAGTCCTTCGTGTTTGATCACGAAAGCACTGGGCATACTATAT
TCAAGTTTGTCATGCAGAAATCTATCCTTGTGCAGTCGCCCAGTGAATTATGTAAAGTTCTTCATGTTTGTACACGAACGCACCGGGTATGCGAGAT
TAAAGTTCATTGAGTGGGGTATATCTTTAAAGTGGGGTTGTACTTTTTCAAAAAAAGTTTCAGTTGACATTGAAATATTTTTCATGCATATGGTTGT
TACCACGGTTATTTAAAAAAATAATCCACTCAAAATAAAGTTCTTCAATTTTTTCAATAATGGTCTTAAATGGCCAGAAAGAAAGTTTCATTTTCAG
GCCAAGATCTCGGCAGTCTCACGGCACAAAATCTCAGGGAAGAAGAAGCCCAGCCAGATCTAATCGGGTGTGTTTCAAATTGTGAAAGCTGGGAAGA
GGAAGTGTGGTGTGTACTACTGCGTCGTAGGTGTAGGTACTACTAGGTAGGAGCGTCAGGGGAGGCAGATACGATGTGACTGTATATTCCTGTATAG
CAAACCGGGTGTTGTTTATTTGGATTTTAAGGCTGCAACCAGTGGCCCACGGCGCGCGGATCCATAGGAAGTAGCAGCGGCAGCGGTACGCGTTGCG
CGCCGAACCGGTTAGTAATAGCCATATTCGCGTGGACGAGCAAGGAGGACGAAATCCTGGCTTCCTACGTCAAGGCCCATGGCGAAGGCAGGTGGAG
GGAGCTCCCCCAGCGAGCTGGTACGTACTAATCCATCGTAATGACGTAATCCCTAGCTAGC
>TaMYB-D7b-2174
ATGGGAGGAGGGCGTGCTGTGCCAAGGAAGGGGTGAAGCGAGGGGCCTGGACGAGCAAGGAGGACGAGATCCTGGCTTCCTACGTCAAGGCCCATG
GCGAAGGCAGGTGGAGGGAGCTCCCGCAGCGAGCTGGTACTACTCGCGAGATGTTATCATGTTAGCTACCGATCCATCGTAAACCGTTCTGGTTCTA
TCCAATGGAGAGCTGGAATGGAAGTGACGCATGCTGCTAACTGAATGAATCTGCATGCAGGTCTGCGGCGGTGCGGCAAGAGCTGCCGGCTGCGGTG
GCTGAACTACCTCCGGCCGAACATCAAGCGGGGCAACATCTCCGACGACGAGGAGGAGCTCATCGTCAGGCTCCACGGCCTGCTCGGCAACAGGTGG
TCCATCATCGCCGGCAGGCTGCCCGGCCGAACAGACAACGAAATCAAGAACTACTGGAACAGCACGCTCGGCAGGAAGGCGCTCCCCGCCCGCCCCG
CCATTGCCGCGGCGAGGACCGTCGCCTCCGGCTCCTCCAGCTCCACGGGGAGCGCCGCGGCGGCGCTGTCTACCTCCGTCCCTGCCGTCCTCCATGG
CGCGGCGCCTTCGTCGCCGGCCGGCGCGGTGTGGGCGCCCAAGCCCGTGAGGTGCACGGGCGGCCTCTTCTTCCGCAGGGAGCAGGAGACGCCGCCG
CCCGCGCCGGTCGTCGAGGAGACGCGGGCCGGAGGAGATCAAGAGGGAGACGCCTGCAGCGGCAGCAGCTCGGAGACGTCGTCGGCCGAGCCGTGCT
CGTCGGGGTCGGGCGGCGGAGACTGGATGGACGACGTGAGAGCCCTGGCGTCGTTCCTGGAGTCCGACGAGGAATGGCTCAAGTCCCTGCACATGGC
CGGTTAA

Supplementary Figure S4 The sequences and locations of two sgRNAs in the *TaMYB7* genes. Two sgRNA sequences are underlined. PAM sequences are in bold and red. The start codon and the splicing sites of the intron are highlighted in yellow and red letters. Exon sequences are highlighted in grey. Apparently, exon 1 in TraesCS7B02G070400 was split into exons due to the insertion of an intron. The intron was caused by two repeated fragments GCGGGACGAG that are indicated in pink.

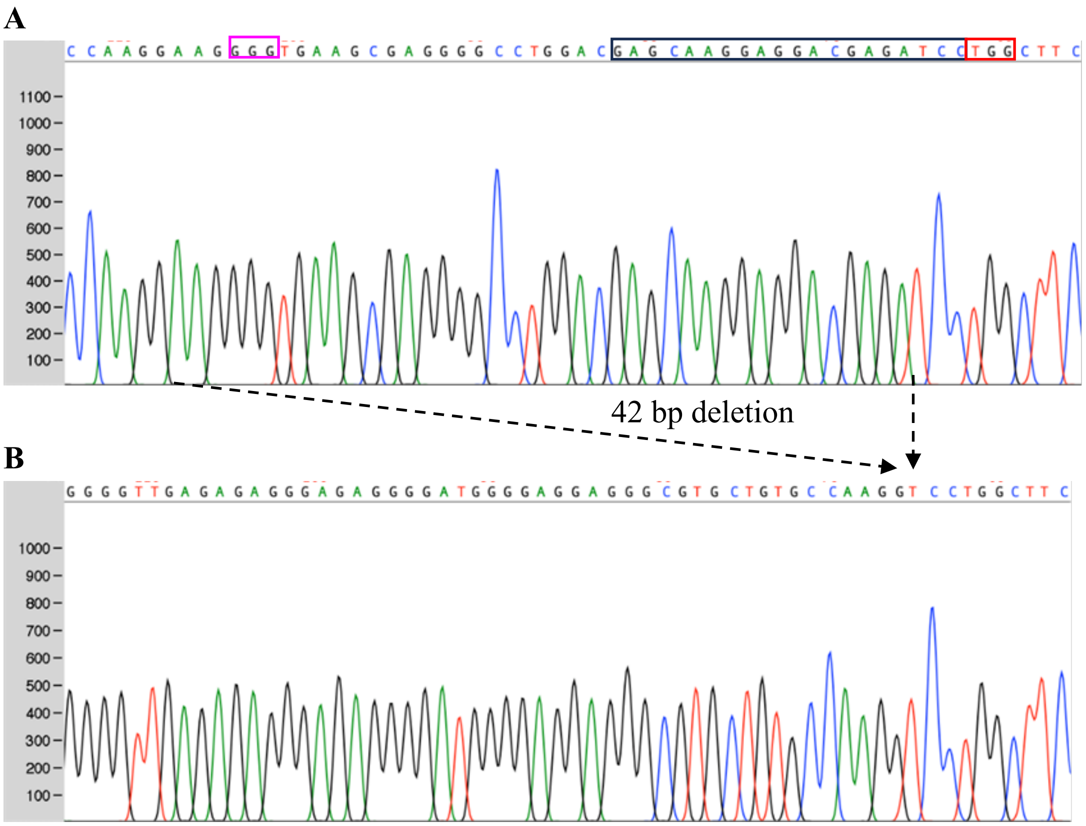

Supplementary Figure S5 The chromatogram sequence of *TaMYB-D7b-ED1*. A, The sequence of wild-type 2174. sgRNA 2 sequence is indicated with a black square. The PAM of sgRNA 2 is indicated with a red square, and the PAM of sgRNA 1 is indicated with a pink square. **B**, *TaMYB-D7b-ED1*. The 42 bp between the two arrows is deleted in *TaMYB-D7b-ED1*.

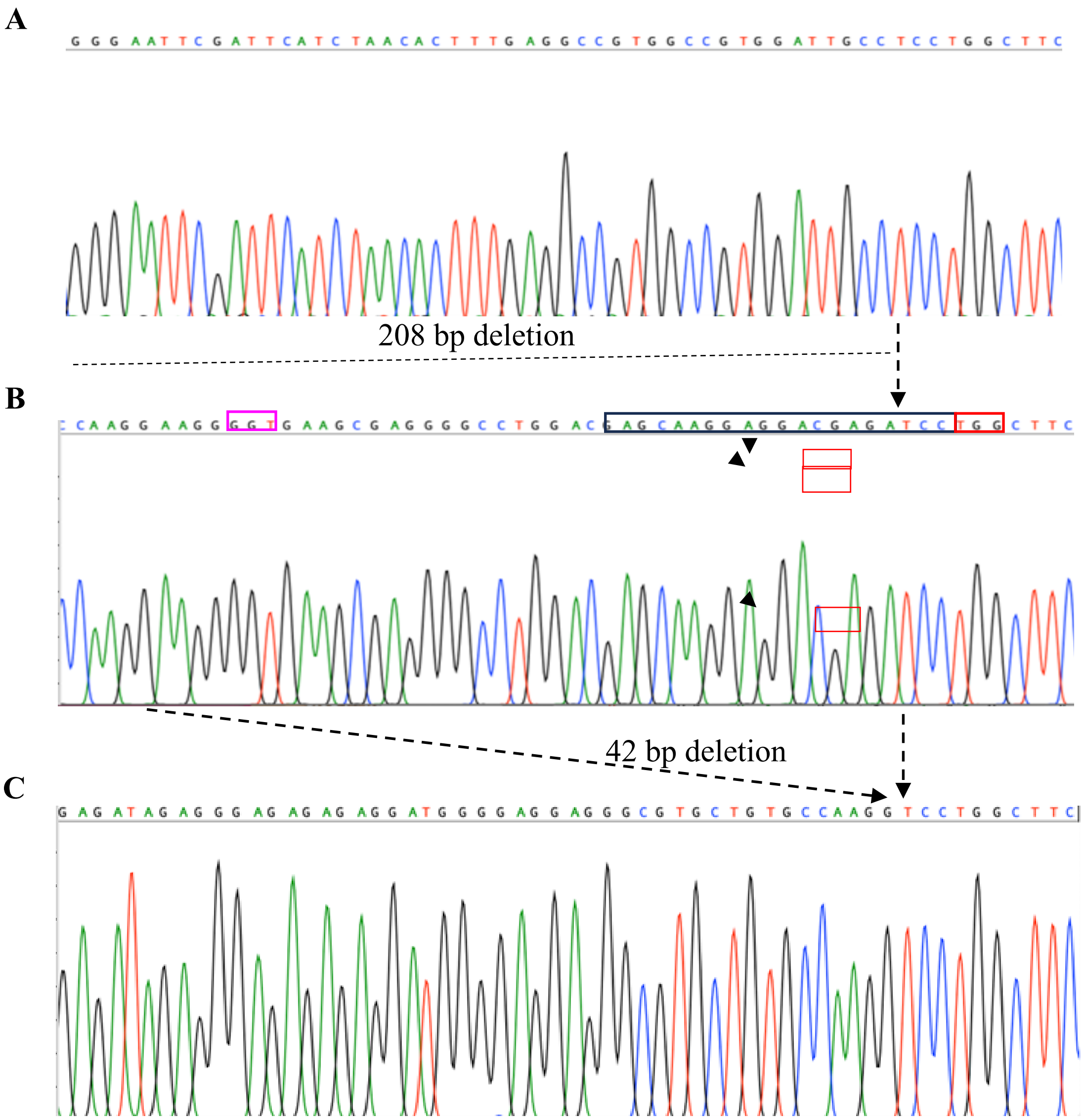

Supplementary Figure S6 The chromatogram sequence of *TaMYB-A7* edited alleles. A, *TaMYB-A7-ED1*. A deletion of 208 bp occurred upstream position 4 from PAM of sgRNA 2. **B**, The sequence of wild-type 2174. sgRNA 2 sequence is indicated with a black square. The PAM of sgRNA 2 is indicated with a red square, and the PAM of sgRNA 1 is indicated with a pink square. **C**, *TaMYB-A7-ED2*. The 42 bp between the two arrows is deleted in *TaMYB-A7-ED2*.

A

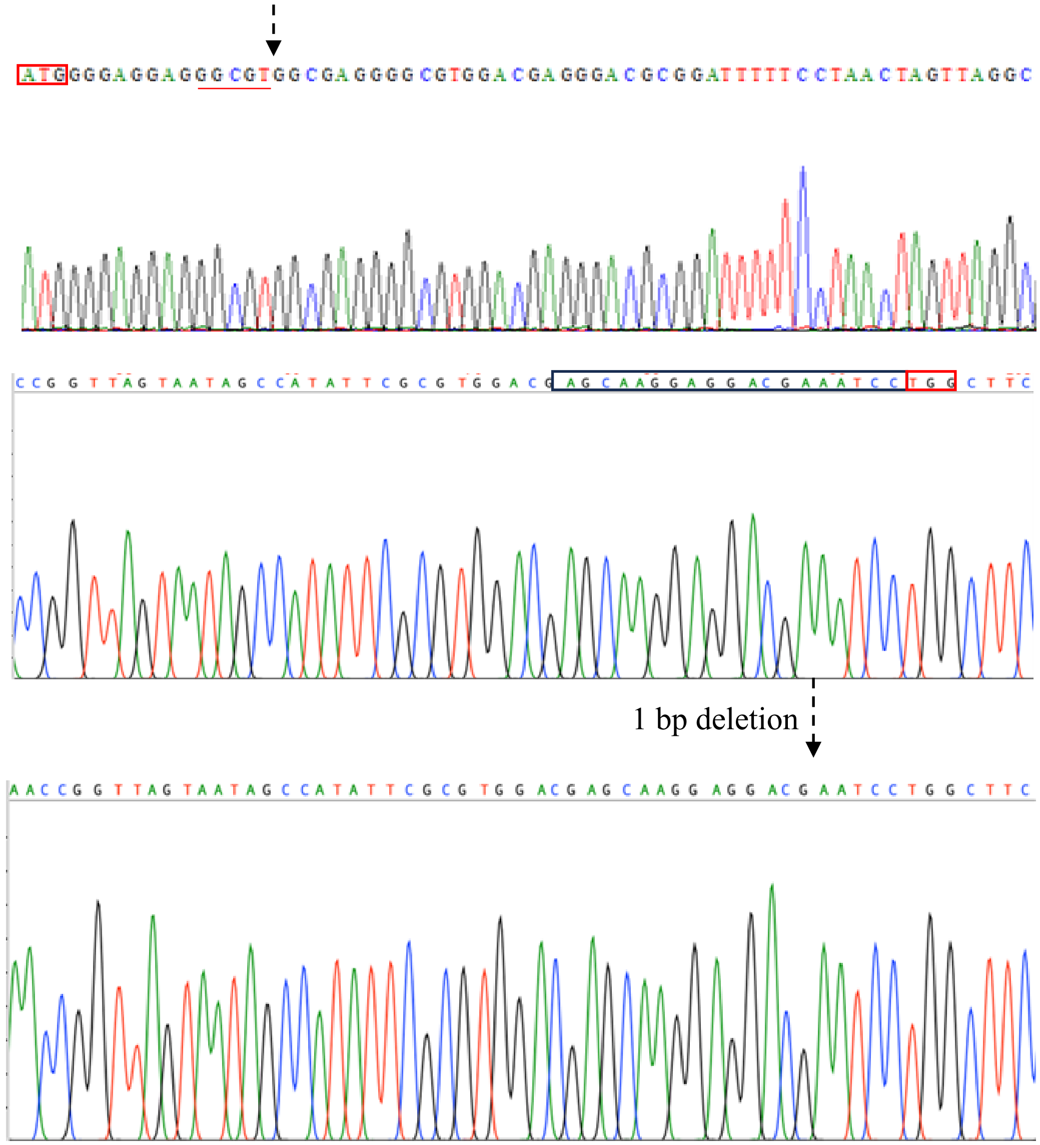

B

C

Supplementary Figure S7 The chromatogram sequence of *TaMYB-B7* edited alleles. A, *TaMYB-B7-ED1*. A 21 bp fragment was deleted in *TaMYB-B7-ED1*. The start codon for translation is indicated with a red square. The partial sgRNA that was not deleted is underlined. **B**, The sequence of wild-type 2174. sgRNA 2 sequence is indicated with a black square. The PAM of sgRNA 2 is indicated with a red square. **C**, *TaMYB-B7-ED2*. The 1 bp, pointed by arrow, is deleted in *TaMYB-B7-ED2*.

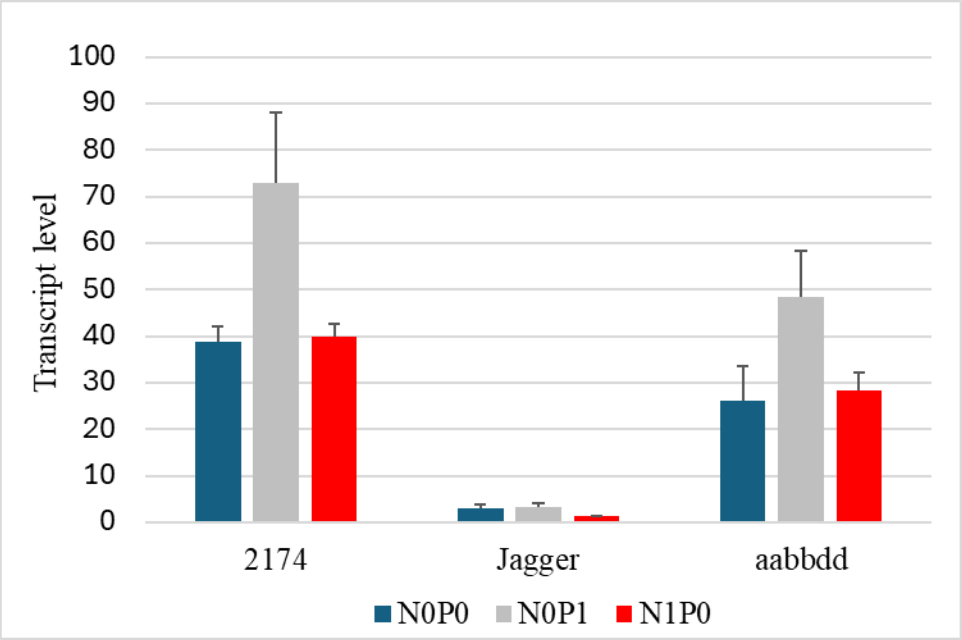

Supplementary Figure S8 The transcript levels of *TaCHSL2* genes in natural and edited mutants of *TaMYB7*. The plants were grown in the commercial soil for two weeks, and N or P fertilizer was supplied into the soils. Leaf samples were collected before the fertilizer (0) or after the fertilizer was added one week (1). Transcript levels were determined by qPCR, with *Actin* as a reference transcript. Values are means ± standard error (*n* = 6).

Supplementary Table S1. N or P content in different treatments in sand culture.

Treatment	Name of the chemical compound	Molecular formula	Molecular mass (g/mol)	Concentration (mg/L)
1. Normal N and P	Calcium nitrate	$Ca(NO)_3$	164.09	820
	monopotassium phosphate	KH_2PO_4	136.09	136
2. Normal N	Calcium nitrate	$Ca(NO)_3$	164.09	820
	monopotassium phosphate	KH_2PO_4	136.09	22.7 (1/6 of normal treatment)
3. Normal P	Calcium nitrate	$Ca(NO)_3$	164.09	136 (1/6 of normal treatment)
	monopotassium phosphate	KH_2PO_4	136.09	136
4. Low N and low P	Calcium nitrate	$Ca(NO)_3$	164.09	136 (1/6 of normal treatment)
	monopotassium phosphate	KH_2PO_4	136.09	22.7 (1/6 of normal treatment)

Supplementary Table S2 Other Macronutrients in sand culture.

Name of chemical compound	Molecular formula	Molecular mass (g/mol)	Concentration (mg/L)
Potassium sulfate	K_2SO_4	174.26	435
Magnesium sulfate	$MgSO_4 \cdot 7H_2O$	246.50	246
Boric acid	H_3BO_3	61.83	2.86
Manganese sulfate	$MnSO_4$-	151.00	1.50
Zinc sulfate	$ZnSO_4 \cdot 7H_2O$	287.55	0.20
Copper sulfate	$CuSO_4$	159.61	0.08
Ferrous sulfate	$FeSO_4 \cdot 7H_2O$	278.10	13.9
Molybdic acid	$H_2MoO_4 \cdot H_2O$	179.94	0.02
Ethylenediamine tetra-acetic acid disodium salt	EDTA	336.21	18.65

Supplementary Table S3. Primers used in this study

Primer name	Primer sequence (5′-3′)	Usage
TaMYB-D7-RT-F2	GCGGCCTCTTCTTCCGCA	Gene expression
TaMYB-D7-RT-R2	CCAGGAACGACGCCAGGG	
CHS-RT-F1	CGTGGAGGAGGTGAGGAAG	Gene expression
CHS-RT-R1	ATCTGCGACTTGTCACACATCC	
TaMYB-A7-ED-F1	CCGGTGTATAAATACCCCAACC	pBUN421 editing
TaMYB-A7-ED-R1	TGCGTTTACGATGGATCAGTACG	
TaMYB-B7-ED-F1	ATTTTAAGGCTGCAACCAGT	Transgenic
TaMYB-B7-ED-R1	GCTAGCTAGGGATTACGTCA	
TaMYB-D7-ED-F1	CCGGTGTATAAATACCCCAGCA	Transgenic
TaMYB-D7-ED-R1	TCGGTAGCTAACATCTCGCGA	
Actin-F2	GGAACTGGCATGGTCAAGGCTG	Expression
Actin-R2	CCCATCCCCACCATCACACC	
TaMYB-D7-dCAP-F1	ATGCAGGTCTGCGGCGGTGC	PCR marker
TaMYB-D7-dCAP-R1	AGGAGCCGGAGGCGACGGTC	
TaMYB-D7-KASP-F1	ACTCGCGAGATGTTATCATGTTAGCTACC	KASP marker
TaMYB-D7-KASP-R1[1]	GAAGGTGACCAAGTTCATGCTCAGCCGGCAGCTCTTGC**T**	
TaMYB-D7-KASP-R2[2]	GAAGGTCGGAGTCAACGGATTCAGCCGGCAGCTCTTGC**C**	

[1]The underlined sequence is the standard FAM tail, while the bold sequence is specific to the Jagger allele.

[2]The underlined sequence is the standard FAM tail, while the bold sequence is specific to the 2174 allele.
